# Deep learning for dual detection of microsatellite instability and *POLE* mutations in colorectal cancer histopathology

**DOI:** 10.1038/s41698-024-00592-z

**Published:** 2024-05-23

**Authors:** Marco Gustav, Nic Gabriel Reitsam, Zunamys I. Carrero, Chiara M. L. Loeffler, Marko van Treeck, Tanwei Yuan, Nicholas P. West, Philip Quirke, Titus J. Brinker, Hermann Brenner, Loëtitia Favre, Bruno Märkl, Albrecht Stenzinger, Alexander Brobeil, Michael Hoffmeister, Julien Calderaro, Anaïs Pujals, Jakob Nikolas Kather

**Affiliations:** 1 Else Kroener Fresenius Center for Digital Health, Medical Faculty Carl Gustav Carus, Technical University Dresden, Dresden, Germany; 2 Pathology, Faculty of Medicine, University of Augsburg, Augsburg, Germany; 3 Department of Medicine I, University Hospital and Faculty of Medicine Carl Gustav Carus, Technische Universität Dresden, Dresden, Germany; 4 Division of Clinical Epidemiology and Aging Research, German Cancer Research Center (DKFZ), Heidelberg, Germany; 5 Pathology & Data Analytics, Leeds Institute of Medical Research at St James’s, University of Leeds, Leeds, United Kingdom; 6 Digital Biomarkers for Oncology, German Cancer Research Center (DKFZ), Heidelberg, Germany; 7 Division of Preventive Oncology, German Cancer Research Center (DKFZ) and National Center for Tumor Diseases (NCT), Heidelberg, Germany; 8 German Cancer Consortium (DKTK), German Cancer Research Center (DKFZ), Heidelberg, Germany; 9 Université Paris Est Créteil, INSERM, IMRB, Créteil, France; 10 Assistance Publique-Hôpitaux de Paris, Henri Mondor-Albert Chenevier University Hospital, Department of Pathology, Créteil, France; 11 INSERM, U955, Team Oncogenèse des lymphomes et tumeurs de la Neurofibromatose 1, Créteil, France; 12 Institute of Pathology, University Hospital Heidelberg, Heidelberg, Germany; 13 Tissue Bank of the National Center for Tumor Diseases (NCT) Heidelberg, Heidelberg, Germany; 14 Medical Oncology, National Center for Tumor Diseases (NCT), University Hospital Heidelberg, Heidelberg, Germany

**Keywords:** Mathematics and computing, Pathology, Diagnostic markers, Diagnostic markers, Tumour biomarkers

## Abstract

In the spectrum of colorectal tumors, microsatellite-stable (MSS) tumors with DNA polymerase ε (*POLE*) mutations exhibit a hypermutated profile, holding the potential to respond to immunotherapy similarly to their microsatellite-instable (MSI) counterparts. Yet, due to their rarity and the associated testing costs, systematic screening for these mutations is not commonly pursued. Notably, the histopathological phenotype resulting from *POLE* mutations is theorized to resemble that of MSI. This resemblance not only could facilitate their detection by a transformer-based Deep Learning (DL) system trained on MSI pathology slides, but also indicates the possibility for MSS patients with *POLE* mutations to access enhanced treatment options, which might otherwise be overlooked. To harness this potential, we trained a Deep Learning classifier on a large dataset with the ground truth for microsatellite status and subsequently validated its capabilities for MSI and *POLE* detection across three external cohorts. Our model accurately identified MSI status in both the internal and external resection cohorts using pathology images alone. Notably, with a classification threshold of 0.5, over 75% of *POLE* driver mutant patients in the external resection cohorts were flagged as “positive” by a DL system trained on MSI status. In a clinical setting, deploying this DL model as a preliminary screening tool could facilitate the efficient identification of clinically relevant MSI and *POLE* mutations in colorectal tumors, in one go.

## Introduction

In colorectal cancer (CRC), microsatellite instability (MSI) is a key biomarker, indicating tumors that are hypermutated and highly immunogenic^1–3^. As a result, CRC patients with MSI are considered as candidates for immunotherapy in both early and advanced stages^4^. Laboratory tests, including polymerase chain reaction (PCR) and immunohistochemistry (IHC), are used for the detection of MSI by identifying a lack in expression of mismatch repair deficiency proteins (dMMR). Since 2019, dozens of academic research studies have shown that Deep Learning (DL) can predict MSI status directly from routine hematoxylin and eosin (H&E) histology slides^5–14^. These studies have led to the regulatory approval of at least one commercial DL-based MSI test in 2022, with several similar products being under development at other commercial entities^14–16^. Although the performance of DL-based MSI tests may exhibit a somewhat reduced specificity compared to gold standard methods, they offer a more rapid and cost-effective means of patient pre-screening at a high sensitivity, reducing the need for validating laboratory tests^14,17^.

Unlike patients with MSI, those with microsatellite stable (MSS) colorectal tumors typically do not respond to immunotherapy. However, there is an exception: those harboring pathogenic mutations in the DNA synthesis proteins DNA Polymerase Epsilon (*POLE*) or DNA Polymerase Delta (*POLD1*). These mutations, specifically in the exonuclease domain of the DNA polymerase family B, disrupt the proofreading function of the DNA polymerase enzyme^18,19^. Consequently, these patients accumulate somatic mutations, resulting in a high immunogenicity, and are likely to respond to immunotherapy^20–22^. Much like CRC with MSI, MSS CRC with *POLE* or *POLD1* mutations also exhibit a response to cancer immunotherapy^22,23^, with ongoing clinical trials for immuno-oncology therapy in such cases^24^. However, these pathogenic mutations are found in only about 1% of CRC patients^25^ and are not typically included in routine screening protocols. As a result, they often remain undiagnosed in clinical practice.

In this study, we aimed to investigate whether DL-based MSI detection methods, which were not explicitly trained to detect *POLE*/*POLD1* tumors, are capable of identifying such cases. Our hypothesis is grounded on research showing that tumors characterized by MSI, and those with pathogenic *POLE*/*POLD1* mutations (but MSS), exhibit shared clinical and morphological characteristics^20,26–29^. Although these tumors are not biologically identical^30^, they share biological features that arise from deficiencies in DNA repair mechanisms, leading to subsequent genomic instability and response to immunotherapy^31–33^. Resulting from these similarities, we hypothesized that the morphological appearance of these tumors in histological H&E images should be comparable enough for DL methods to detect them, even if trained solely on detecting MSI. This would be clinically valuable, as there is currently a lack of rapid, cost-effective and widely accessible methods to diagnose CRC cases with pathogenic *POLE/POLD1* mutations.

## Results

### Prediction of MSI status from histological data via Deep Learning across three external cohorts

**Fig. 1:**
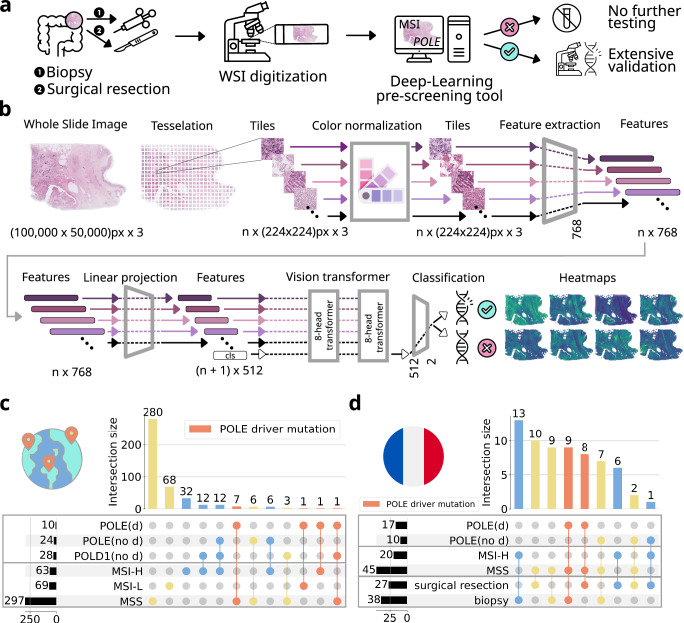
Experimental design, study overview and cohort characterization. **a** A suspected case of colorectal cancer (CRC) prompts a biopsy or surgical resection to obtain a tissue sample. This sample is then digitized into a Whole Slide Image (WSI) for analysis by a clinical Deep Learning system, which has the potential to pre-screen for MSI and *POLE* cases, pending external validation and regulatory approval as a medical device. **b** Our Deep Learning pipeline starts with tessellation of Whole Slide Images (WSIs) into smaller, relevant tiles while discarding non-informative background areas. We then extract n feature vectors from n color-normalized tiles, which range in size from 100,000 to 50,000 pixels across three color channels. These vectors are compressed into a more compact feature space and processed using a two-layer, eight-head Vision Transformer (ViT) architecture. Within this system, a ‘class token’ is simultaneously trained to generate the final MSI prediction. To aid pathological evaluation, we create heatmaps that visualize the areas of focus determined by the ViT’s attention mechanisms. **c** Molecular characterization of the TCGA (The Cancer Genome Atlas) cohort with respect to MSI and MSS (MSI-L/MSS). Combinations of microsatellite status and *POLE*/*POLD1* (“d”: driver) mutations are shown (orange: *POLE* driver mutation, blue: MSI-H, yellow: MSI-L/MSS). **d** Molecular characteristics of the APHP (Assistance Publique–Hôpitaux de Paris, resection and biopsy) cohorts with respect to MSI and MSS cases. Combinations of microsatellite status and *POLE*/*POLD1* (“d”: driver) mutations are shown (orange: *POLE* driver mutation, blue: MSI-H, yellow: MSS). The icons on all panels are obtained from www.flaticon.com.

**Fig. 2:**
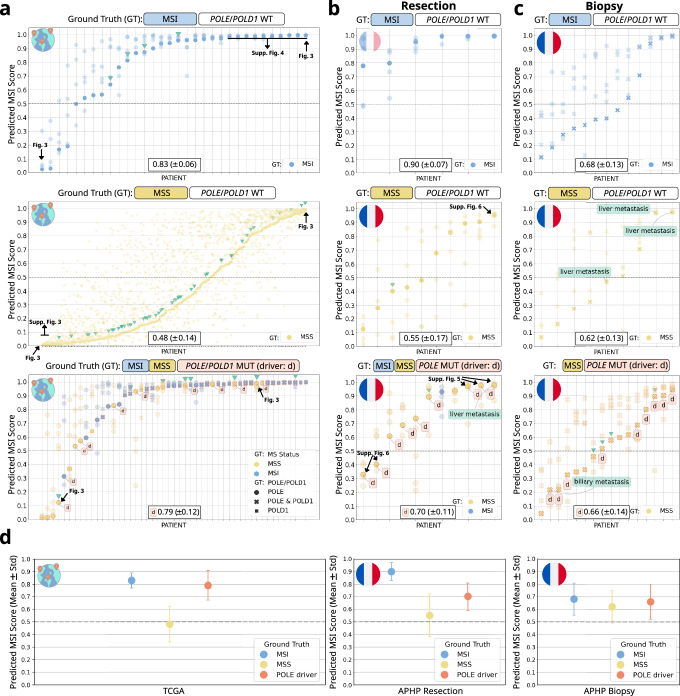
Results of MSI and *POLE* prediction experiments with Vision Transformer based pipeline. Results are shown for external validation on TCGA (The Cancer Genome Atlas, International) and APHP Resection and Biopsy (Assistance Publique–Hôpitaux de Paris, France). Each patient is shown with 5 dots representing their prediction scores from 5-fold cross-validation. Highlighted dots correspond to the median AUROC model based on the TCGA cohort. The classification threshold is set to 0.5 for all cohorts. Mean and standard deviation of the predicted MSI score are shown, with values computed for driver mutations only in case of *POLE*/*POLD1*. Microsatellite status is indicated by color, wild type by WT, mutated by MUT, driver mutations by “d”, liver metastasis samples by green boxes and early-onset colorectal cancer patients (age at diagnosis <50 years)^58^ by a green triangle. Arrows point to the corresponding heatmaps of selected samples. **a** In the TCGA testing cohort, prediction scores were calculated for patients with MSI (excluding *POLE*/*POLD1* mutations, top chart), MSS (excluding *POLE*/*POLD1* mutations, middle chart) and *POLE*/*POLD1* mutations (bottom chart). MSS group includes MSI-L and MSS. **b**, **c** In APHP resection (**b**) and biopsy (**c**) testing cohort, prediction scores were calculated for patients with MSI (excluding *POLE*/*POLD1* mutations, top chart), MSS (excluding *POLE* mutations, middle chart) and *POLE* (bottom chart). **d** The mean and standard deviation for Predicted MSI Scores in panel **a**–**c** based split are calculated for the cohort and ground truth regarding microsatellite and *POLE*/*POLD1* status. For *POLE*/*POLD1* mutated cases, only driver mutations are considered. The icons indicating the origin of the cohorts are sourced from www.flaticon.com.

Digital whole slide images of H&E stained histopathology tissue of CRC cases were collected from four patient cohorts (Fig. 1a and Supplementary Table 1): DACHS (Darmkrebs: Chancen der Verhütung durch Screening, *N* = 2039)^34,35^, TCGA (The Cancer Genome Atlas, *N* = 429, Fig. 1c), APHP (Assistance Publique–Hôpitaux de Paris/Public Assistance Hospitals of Paris) surgical resection (*N* = 27, Fig. 1d) and APHP biopsy (*N* = 38, Fig. 1d). The cases from the DACHS cohort were then used to train a transformer-based Deep Learning model (Fig. 1a, b) for the prediction of MSI status. We assessed the model’s performance through patient-level cross-validation. Our findings revealed a state-of-the-art Area under the Receiver Operating Curve (AUROC) of 0.94 ± 0.03 (*p* = 0.00, Supplementary Fig. 2a) for MSI prediction, reiterating that MSI status can be reliably predicted from histopathology (Supplementary Fig. 2b). We used a pre-defined threshold of 0.5 to binarize outcomes based on the prediction scores obtained from the output neurons of the classifier network, with cases above and equal to 0.5 classified as positive, and those below as negative. This resulted in a sensitivity of 0.87 ± 0.07 (mean ± standard deviation), a specificity of 0.88 ± 0.04, a negative predictive value (NPV) of 0.98 ± 0.01 and a positive predictive value (PPV) of 0.47 ± 0.08. The distribution of prediction scores within the DACHS cohort indicates accurate classification for most MSI and MSS cases, including those patients under 50 years of age (Supplementary Fig. 2). These observations highlight the model’s robust performance on internal data, underscoring the need for external validation. To address this, we tested the classifier’s generalizability by utilizing three external cohorts (Fig. 2a–c) with previously unseen data. For the TCGA cohort, consisting of *N* = 429 patients, an AUROC of 0.87 ± 0.02 (*p* = 0.00, Supplementary Fig. 2a) was achieved. Furthermore, our model reached a sensitivity of 0.93 ± 0.02, a specificity of 0.51 ± 0.10, a NPV of 0.98 ± 0.00 and a PPV of 0.25 ± 0.03 in the TCGA cohort. The mean prediction scores (± standard deviation) in this cohort were 0.83 ± 0.06 for MSI patients, 0.48 ± 0.14 for MSS patients, and 0.79 ± 0.12 for those patients harboring pathogenic *POLE* or *POLD1* mutations (Fig. 2a, d).

The APHP resection and biopsy cohorts were designed to include a nearly equal number of patients with MSI and MSS, ensuring a balanced representation between the two groups. Within the APHP resection cohort, 5.60 ± 0.49 (mean ± standard deviation) of 6 MSI patients were accurately predicted to be MSI. Conversely, 4.60 ± 0.80 out of 10 MSS patients were correctly categorized, with the remaining ones falsely predicted to be MSI. This resulted in a sensitivity of 0.93 ± 0.08, specificity of 0.46 ± 0.08, PPV of 0.51 ± 0.03, and NPV of 0.93 ± 0.08 (Fig. 2b), which is similar to commercially available methods for detection of MSI from H&E slides^14^. The mean MSI prediction score was 0.90 ± 0.07 for MSI patients, 0.55 ± 0.17 for MSS patients, and 0.70 ± 0.11 for patients with pathogenic *POLE* mutations (Fig. 2d). This implies that our model predicts MSI status on par with a commercially available assay and is a viable pre-screening tool.

For the APHP biopsy cohort, 9.20 ± 2.40 of the 13 MSI patients were accurately predicted to be MSI, while 5.80 ± 1.60 out of 9 MSS patients were incorrectly predicted to be MSI. This led to a sensitivity of 0.71 ± 0.18, specificity of 0.36 ± 0.18, PPV of 0.61 ± 0.05, and NPV of 0.47 ± 0.12 (Fig. 2c). Of note, we found that two cases from the last group which were falsely predicted as MSI, originated from liver metastases (Fig. 2c, middle) as opposed to primary tumors like all cases in the training set, indicating that the system’s performance on liver biopsy tissue could be limited. The mean MSI prediction scores for this cohort were: 0.68 ± 0.13 for MSI patients, 0.62 ± 0.13 for MSS patients, and 0.66 ± 0.14 for patients bearing pathogenic *POLE* mutations (Fig. 2d).

To conclude, our transformer-based Deep Learning approach demonstrated robust capabilities in predicting MSI status from pathology slides of surgical resection samples. Next, we assessed the predictions of this classifier in patients harboring *POLE* driver mutations.

### Deep Learning for MSI classification also identifies *POLE* driver mutants in three external cohorts

We hypothesized that MSS tumors harboring *POLE* driver mutations share a common phenotype with MSI tumors. To investigate this, we obtained three cohorts with *POLE* sequencing data: TCGA, the APHP surgical resection and the APHP biopsy cohort. In TCGA, *N* = 10 tumors had *POLE* driver mutations (Fig. 1c and Supplementary Table 2), reflecting the natural prevalence of these rare mutations in CRC. The APHP resection cohort was composed of *N* = 8 *POLE* driver mutants, *N* = 6 MSI and *N* = 12 MSS patients, whereas the APHP biopsy cohort was composed of *N* = 9 *POLE* driver mutants, *N* = 13 MSI and *N* = 8 MSS patients (Fig. 1d, Supplementary Table 3). None of the three external cohorts included *POLD1* driver mutation cases.

We observed that 9.00 ± 0.63 out of 10 *POLE* driver mutant patients in the TCGA cohort were predicted as MSI. The two *POLE* driver mutant cases which were not classified as MSI by the median model (Fig. 2a, bottom) carried the *POLE* mutations S459F and V411L, which are considered pathogenic^29,36–39^. Specifically, TCGA-AG-A002 had mutations S459F (d) and R150* and a median prediction score for MSI of 0.12, while TCGA-AA-A00N carried V411L (d) and L1255V and had a median MSI prediction score of 0.31 (Supplementary Table 2). Overall, there was a discernible trend towards high MSI prediction scores for the majority of POLE/POLD1 mutant samples. For scores in the range of 0.8 and above, there was reduced variation among the model’s predictions, as opposed to the transitional range (Fig. 2a bottom). Furthermore, it can be noted that for the two misclassified cases in TCGA based on the model with the median AUROC, the majority of the 5 deployed models predicted them to be as MSI when the 0.5 threshold was applied.

In the APHP resection cohort, 6.00 ± 0.63 out of 8 *POLE* driver mutant patients were predicted to be MSI (Fig. 2b, bottom). We further evaluated the performance of our model on biopsy samples, which are known to be inherently difficult to assess by DL-models^40^. For the APHP biopsy cohort, 6.40 ± 0.80 out of 9 *POLE* mutant patients were predicted to be MSI (Fig. 2c, bottom). Just as in the MSI cases, we observed that there is less uncertainty regarding *POLE* mutations in the APHP resection cohort compared to the APHP biopsy cohort (Fig. 2b, c, bottom). These findings confirm that the analysis of biopsies using DL is more challenging compared to that of surgical resection slides due to inconsistent sample size, quality, and tumor content.

Understanding the pathogenicity and biological relevance of *POLE* mutations is still an evolving field^41,42^. Hotspot mutations show a restriction depending on the tumor type^43^, with *POLE* p.P286R mutations being of particular relevance in CRC^43,44^. For predicting *POLE* p.P286R mutations, our model reached a comparable or even better prediction scores (mean ± standard deviation) than calculated for all driver mutations in TCGA (0.96 ± 0.04), APHP resection (0.70 ± 0.29) and APHP biopsy (0.73 ± 0.16), indicating that our algorithm is able to identify morphological features associated with this particular CRC-specific *POLE* mutation. Taken together, our findings demonstrate that DL-based MSI detection methods possess the capacity to identify *POLE* mutants. This provides compelling evidence for a common morphological phenotype between MSI and *POLE* mutations in CRC, thereby confirming our initial hypothesis.

### Explainability of the morphological patterns associated with MSI and POLE mutations

**Fig. 3:**
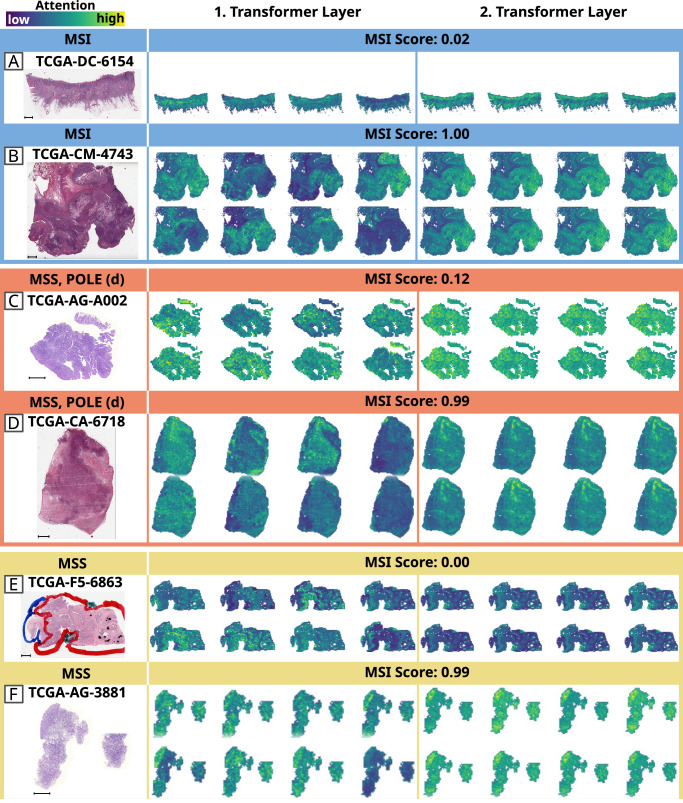
Attention heatmaps of selected samples from the TCGA (The Cancer Genome Atlas) cohort. For each group with ground truth MSI (blue, **A**, **B**), MSS & *POLE* mutated (orange, **C**, **D**), and MSS (yellow, **E**, **F**) we chose a sample with a high and a low MSI prediction score, respectively. The associated whole slide images can be viewed under the GDC Data Portal: https://portal.gdc.cancer.gov/. Heatmaps were derived from a model that showcases the median AUROC among 5 trained models for this cohort. Due to the transformer’s architecture, which consists of 2 layers and 8 heads, each slide yields 16 distinct heatmaps. Blue regions signify areas with low attention, while yellow designates high attention areas critical for predictions. The scale bars indicate a length of 2 mm. **A**, **C**, **E** illustrate low MSI-prediction scores, characterized by adenocarcinoma NOS (not otherwise specified) histology, the presence of dirty necrosis, and a reduced number of tumor-infiltrating lymphocytes. **B**, **D**, **F** demonstrate high MSI prediction scores, associated with morphological hallmarks such as abundant extracellular mucin (seen in **B** and **F**), a medullary growth pattern (visible in **D**), and an enhanced presence of tumor-infiltrating lymphocytes.

Having established that a morphology-based classifier trained on MSI tumors can also identify *POLE* mutant tumors, we sought to determine the distinct morphological patterns associated with these tumors. In order to visualize the distribution of attention at a slide-level, we conducted an analysis using attention heatmaps for samples in TCGA (Fig. 3) and DACHS (Supplementary Fig. 3a). Specifically, we examined samples with the highest and lowest prediction scores across all ground truth classes, including MSI, MSS, and *POLE* driver mutations (*POLE* only for TCGA, regardless of MS status). Here, we observed that high-attention areas included almost exclusively tumor tissue, whereas other tissue types, such as regular tumor-free intestinal mucosa or uninvolved pericolonic/mesorectal adipose tissue, were not highlighted. The heatmaps also reveal that the model seems to be quite robust against pen markings, since prevalent pen markings are not highlighted in the majority of cases (Supplementary Fig. 3). The results depicted in the figures also indicate that the eight attention maps of the first transformer layer show more diverse attention patterns than in the second transformer layer. For two samples (Supplementary Fig. 3b), attention within the transformer varies significantly among different attention heads: some focus on larger regions, while others concentrate on smaller areas. This variance becomes more uniform in the second transformer layer (Fig. 3, Supplementary Fig. 3). Furthermore, we revised the histomorphology of the cases with lowest (*N* = 10, heatmaps in Supplementary Fig. 4) and highest (*N* = 10, heatmaps in Supplementary Fig. 5) MSI prediction scores within TCGA with regards to histology, tumor-infiltrating lymphocytes (TILs) and the presence of dirty necrosis (Supplementary Table 5). High MSI prediction scores were significantly associated with the presence of extracellular mucin or a medullary growth pattern (*p* < 0.001), higher numbers of TILs (*p* = 0.002) and the absence of dirty necrosis (*p* = 0.002) (Supplementary Table 6), which is in line with the characteristic phenotype Shia et al. have already described in their comprehensive histomorphologic characterization of *POLE* mutant CRCs^27^. In the APHP resection cohort, prediction and attention heatmaps for selected samples have been delineated in Supplementary Figs. 6-7. Within these figures, a specific MSS patient sample, taken from a liver metastasis, was classified as MSI (refer to Supplementary Fig. 6A). Additionally, two MSS patients with pathogenic mutations in *POLE*, both of whom were classified as MSI, are presented in Supplementary Fig. 6B-C. These findings lend considerable weight to the hypothesis of this study. In contrast, the sole two MSS patients with pathogenic mutations in *POLE*, who were classified as MSS, are shown in Supplementary Fig. 7A-B, accompanied by another MSS case which was incorrectly predicted as MSI (Supplementary Fig. 7C). To gain further insights into morphological features associated with our predictions, we qualitatively reviewed another ten misclassified TCGA MSS cases which were *POLE* wild type (WT) but were assigned high MSI prediction scores by our model. Three of these cases were consistent with the diagnosis of mucinous adenocarcinoma (≥50% extracellular mucin content), and one case showed a partly mucinous differentiation (~40% extracellular mucin content). The other cases showed gland-forming and cribriform architecture (adenocarcinoma, non otherwise specified, low-grade), but all cases had a relatively high tumor-to-stroma ratio, resembling a solid/medullary growth pattern, and also partially showing high number of TILs (Supplementary Table 7). All of these morphological features are known to be associated with MSI, hence they are plausible confounding factors^26,27,45^. Thus, the misclassification of the model is reasonable as it reflects the categorization a pathologist would likely make. Combining these results, it is clear that the morphological patterns detected through our method provide strong validation of our study’s underlying hypothesis.

## Discussion

The advent of cancer immunotherapy has fundamentally changed the treatment landscape in oncology, notably in tumor types such as lung cancer and melanoma where immune checkpoint inhibitors elicit prolonged responses even in metastatic cases^46,47^. Given the current advance in medical treatments, immunotherapy in neoadjuvant and adjuvant settings is increasingly utilized, promising potential for improved response rates across different cancer entities. Despite being one of the most common types of cancer, CRC is notoriously unresponsive to immunotherapy. Only a small fraction (<10% in metastatic stages^48^) of CRC cases, specifically MSI tumors, benefit from immunotherapy^49^, leading to its use even in the neoadjuvant setting, and necessitating upfront MSI testing in all CRC patients. Several techniques for MSI detection include IHC, PCR, next-generation sequencing (NGS)-based testing (via computational methods such as MSIsensor or MANTIS^50^) and a DL-based assay that predicts MSI status via the analysis of digital images of H&E stained histopathology slides (MSIntuit^TM^, Owkin, France^14^). Among the majority of non-MSI CRC patients, a rare, immunotherapy-responsive subgroup exists with *POLE* or *POLD1* mutations, affecting fewer than 1% of CRC cases but highly significant for impacted individuals. Identifying these mutations, however, involves expensive genetic testing, not routinely conducted in CRC cases nor widely available.

Our study demonstrates the effectiveness of a DL method in MSI screening, capable of identifying not only MSI but also *POLE* mutant tumors, classifying them as MSI. This model, initially trained on CRC data to differentiate MSI from MSS cases, utilizes specific characteristics in H&E images for this purpose. The ability of our DL model to detect *POLE* mutations — typically identified via extensive genetic data acquisition and processing — represents a major breakthrough, suggesting our model’s broader utility beyond MSI detection alone. Despite the challenge in conclusively determining these features within our current technological framework, tools like heatmaps assist in correlating visible characteristics with established pathological patterns, indicating that our model captures features common to both MSI and mutations recognizable by trained pathologists. Therefore, describing our model solely as an MSI detector would be an oversimplification as it effectively identifies various mutation patterns, highlighting its diagnostic capabilities beyond initial expectations. This evidence points to a common histopathological profile shared by MSI and *POLE* mutations, allowing a DL system trained on MSI alone to reliably detect *POLE* mutations. The overlapping morphological features between MSI and many *POLE* tumors, such as high levels of tumor-infiltrating lymphocytes, a medullary growth pattern, substantial mucin production, and the absence of dirty necrosis^20,26–29^, are not novel findings. Interestingly, some of these features seem to be site-agnostic and also already known for *POLE* mutant endometrial carcinomas^51^. The assessment of attention heatmaps in our study reinforced the foundational morphological attributes critical to our DL approach. Our analysis not only corroborated our initial observations with respect to the MSI classifier but also underscored the model’s resilience to artifacts like pen markings.

Our model’s categorization of *POLE* cases as MSI, while not technically predicting *POLE*, is still clinically significant. Both MSI and *POLE* mutations, associated with hypermutation^52^, increased immune response, and better outcomes with immunotherapy^20,22,52^, present valuable targets for treatment. Hence, deploying DL screenings could help in identifying potential *POLE* mutation carriers clinically. In practice, after thorough external validation and the necessary regulatory approvals, the model could help in ruling out *POLE* and MSI mutations in samples classified as MSS. This serves as a similar approach to existing DL-based MSI testing tools used for preliminary screening. Following an MSI-positive result from our model, a cost-effective IHC test could be conducted to detect dMMR, correlating with recommendations for existing DL products^14^. The detection of dMMR suggests MSI presence. Conversely, an MSI prediction with proficient MMR necessitates further analysis. According to our findings, in CRC cases of proficient MMR, *POLE* mutations might be the underlying cause for an MSI-like prediction. In such scenarios, it is advised to use cost-effective Sanger sequencing for *POLE* mutations or small NGS panels covering *POLE*/*POLD1* mutations. This is particularly recommended for young male patients with right-sided tumors, as these clinical-pathological features are often associated with *POLE* mutations^20^. Adopting this tiered testing strategy could significantly reduce the need for broad, expensive panel sequencing for *POLE* mutations, thereby streamlining and providing more cost-effective clinical workflows. This becomes increasingly pertinent with the anticipated incorporation of Immuno-oncology (IO) therapies for *POLE* mutant CRCs into routine clinical management.

Technically, our study enhances a robust, previously established DL pipeline^53^ with some innovative elements. For final predictions, we employed an ensemble of 5 models, each trained on distinct 80% subsets of the DACHS training cohort. This ensemble approach generates a consensus indication among the models, which can be interpreted as a measure of confidence in the predictions. Our findings indicate that the models are more confident in their predictions when the scores are markedly high or low, as opposed to those around the mid-range (0.5 ± 0.3). This trend is especially evident in MSI or *POLE* mutation cases derived from surgical resections in the TCGA and APHP datasets. Conversely, there is more uncertainty in predictions for biopsies and MSS cases. Further investigation into the learning mechanics of the model and understanding the elements influencing these extreme prediction values and the origins of uncertainty could lead to further refinement of the model. Our future research should focus on leveraging this methodology to quantify the clinical relevance of ensemble-based prediction uncertainty, ideally incorporating additional cohorts with *POLE* mutant samples. We suggest that data on therapeutic outcomes should be collected in future patient cohorts.

Our research is subject to some constraints, predominantly the limited number of *POLE* mutants and significant class imbalance impacting statistical analysis. Notably, many DL studies in CRC use the TCGA cohort, but it includes only ten relevant *POLE* driver mutants and lacks *POLD1* driver mutants. Despite the inaccessibility of *POLD1* mutant samples, our study sought to solidify our results’ robustness and general applicability by incorporating a considerable number of *POLE* mutation cases from a major French reference center (APHP). It is pertinent to note that the prevalence of *POLE* testing in CRC remains limited, impacting the sample size of *POLE* mutations in our study, although their significance is only just being recognized. Our dataset included 28 *POLD1* mutants and 61 *POLE* mutants, 27 of which were *POLE* driver mutations (Fig. 1c, d). The APHP cohort, which comprised two sub-cohorts of surgical resection specimens and biopsies, respectively, confirmed the generalizability of our findings. Consistent with prior research^8^, our results revealed lower performance on biopsy samples, suggesting current DL approaches are more effective with surgical specimen analysis. However, as biopsy-based testing is becoming more common, it is important to develop algorithms that can effectively process these samples in the future^4^. Additionally, the total sample number in the APHP cohort complicates the interpretation of MSI and MSS, with the liver metastasis cases being the most challenging. Results from DL models, trained on primary tumors but applied to metastatic tissues, often demonstrate unsatisfactory discriminative performance^54^. This becomes apparent in the distinct scatter of the model predictions for MSS cases. To address these challenges, further studies could explore how the selection criteria for defining final predictions impact outcomes. For example, instead of basing the final prediction on the median fold guided by the AUROC, it might be more effective to consider the majority of predictions from the 5 folds. Investigating this approach could lead to a more refined method of setting an optimized threshold for binarization. However, adjusting the threshold for specific cohorts would limit generalizability and would ideally require more data. The goal would be to encompass a greater number of MSI and *POLE* cases while more accurately classifying MSS, thereby minimizing the incidence of falsely predicted cases lacking *POLE*/*POLD1* driver mutations.

In conclusion, our study demonstrates the capability of a DL screening tool, initially trained for MSI classification, to extend its utility beyond identifying MSI status in patients. Notably, it can also discern MSS CRCs harboring *POLE* mutations. This finding implies a shared histopathologic phenotype between MSI and *POLE* mutations, which a DL system, focused solely on MSI, can effectively detect. As we move forward, such technology might play a pivotal role in the preliminary screening for *POLE* mutations within MSS CRCs, potentially identifying a small yet significant patient group for targeted treatment strategies. Our study underscores the importance of collecting these rare samples, as the significance of these mutations is becoming increasingly evident. Nevertheless, it is paramount to further validate and couple these DL-based screenings with subsequent genetic confirmation to bolster diagnoses. The ability to accurately and efficiently identify CRC patients with pathogenic *POLE* gene mutations could greatly enhance both diagnostic processes and therapeutic outcomes in this field.

## Methods

### Patient samples

**Fig. 4:**
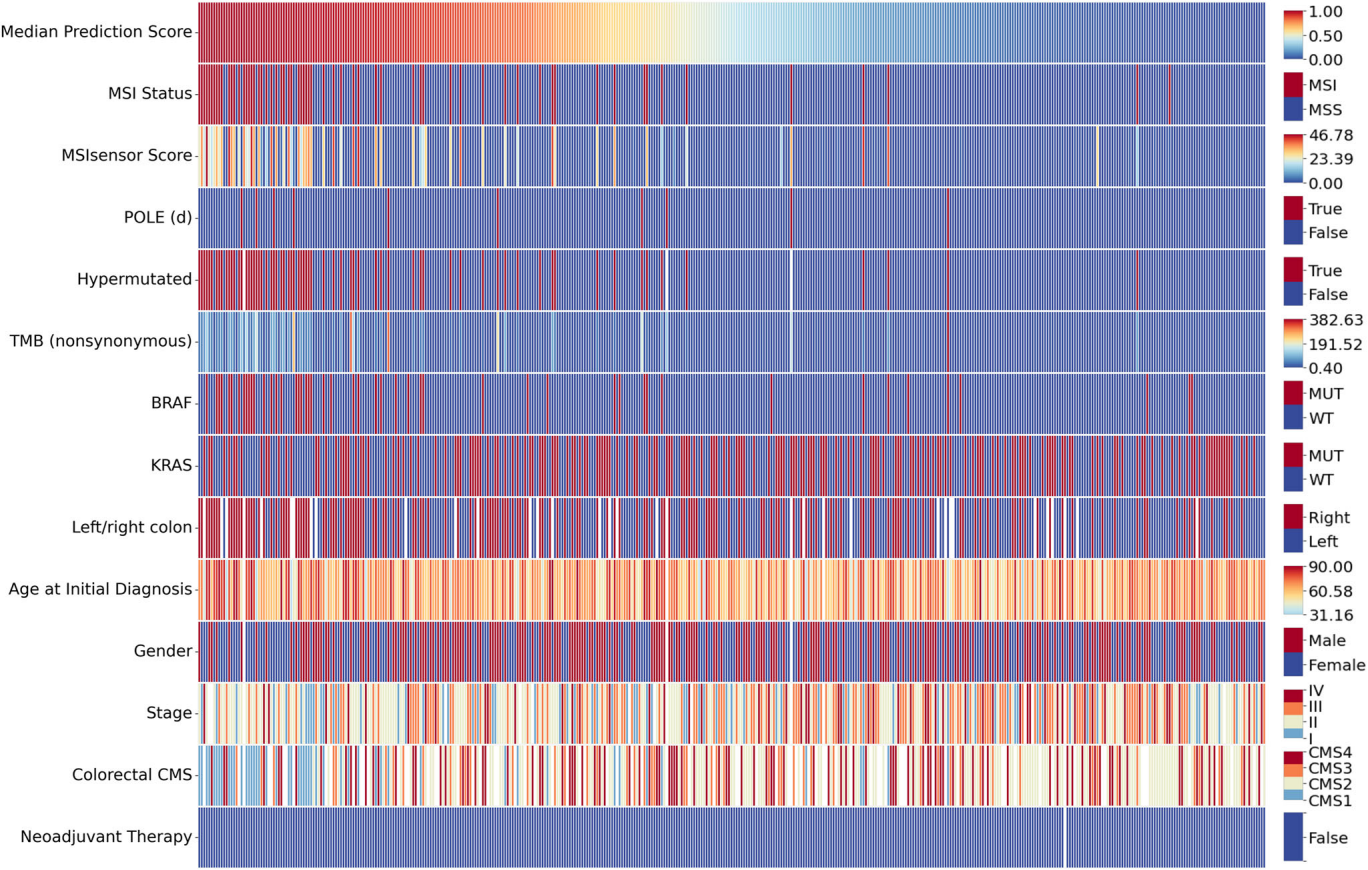
Selected molecular characteristics for the TCGA (The Cancer Genome Atlas) cohort. The columns correspond to individual patients and are sorted by “Median Prediction Score”, representing the median model of 5-fold cross-validation evaluated using AUROC. “MSI Status” is the ground truth of microsatellite status. *POLE* (d) denotes patients with a *POLE* driver mutation. TMB represents tumor mutational burden. For BRAF and KRAS “MUT” indicates the presence of the respective mutation and “WT” the absence of the mutation (wild type). “Stage” ranges from one to four and indicates the cancer stage. The same range is used by “Colorectal CMS” which stands for the consensus molecular subtypes (CMS) of colorectal cancer. Empty cells represent cases where the information regarding specific characteristics is missing.

In this study we used the following independent patient cohorts (Supplementary Table 1): DACHS (Darmkrebs: Chancen der Verhütung durch Screening, Southwest Germany, *N*_all_ = 2039, *N*_MSI_ = 210 (10.3%))^34,35^, TCGA (The Cancer Genome Atlas, *N*_all_ = 429, *N*_MSI_ = 63 (14.7%)), APHP (Assistance Publique–Hôpitaux de Paris/Public Assistance Hospitals of Paris) resection (*N*_all_=27, *N*_MSI_ = 7 (25.9%), Fig. 1d) and APHP biopsy (*N*_all_=38, *N*_MSI_ = 13 (34.2%), Fig. 1d). DACHS is a population-based case-control and patient cohort study on CRC including samples from patients of all tumor stages (I-IV) collected from different laboratories in the south-west of Germany coordinated by the German Cancer Research Center (Heidelberg, Germany). The APHP cohorts are consecutive case series of a total of *N* = 27 *POLE* mutant, with *N* = 17 *POLE* driver mutant, colorectal cancers collected between 2015 and 2023, as well as a matched cohort of *N* = 19 MSI and *N* = 20 MSS tumors from the same pathology archive. In this cohort the control tumors (MSI and MSS tumors) underwent *POLE* testing and are proven to be *POLE* WT. TCGA is the public repository “The Cancer Genome Atlas”, available at https://portal.gdc.cancer.gov/, USA^55,56^, which includes colorectal cancer of any stage. The microsatellite status for the TCGA samples was determined by Polymerase Chain Reaction (PCR) testing^57^. Molecular features of DACHS, TCGA, APHP surgical resection and biopsy cohorts are shown in Fig. 1c, d (Supplementary Table 1). Sociodemographic and relevant clinical features are shown in Supplementary Table 1. Additional molecular information is presented in Fig. 4 for TCGA and in Supplementary Fig. 1 for DACHS and APHP. A comprehensive list with molecular characteristics of patients with *POLE/POLD1* mutations can be found in Supplementary Table 2 for TCGA and Supplementary Table 3 for APHP. As shown in the tables, this study does not include any *POLD1* driver mutations. Therefore, only *POLE* may be referenced in parts of the following sections. The distribution of MSI status was available for all cohorts (Supplementary Table 1). Details on the methods used for genetic testing of patients in these cohorts are given in Supplementary File 1: Supplementary Methods. Cases of early-onset colorectal cancer (EO-CRC) with patient age under 50 at point of diagnosis are highlighted in the results^58^. This retrospective analysis of scanned images of anonymized tissue samples from various cohorts of cancer patients was conducted in accordance with the Declaration of Helsinki. The data were collected, anonymized, and ethical approval was obtained. The use of tissue samples from DACHS was approved by the ethics committees of the Medical Faculty at Heidelberg University (310/2001) and the state medical boards of Baden-Wuerttemberg and Rhineland-Palatinate^59^. All participants the DACHS study provided written informed consent for the scientific analysis of their data and samples. The use of tissue samples from the APHP cohort was approved by the local ethics committee of Henri Mondor University Hospital (IRB N° 00011558 ; 2021-123). For APHP and TCGA, there was no informed consent required by local regulations for a retrospective analysis of anonymized data. The overall analysis was approved by the ethics board of the Medical Faculty of Technical University Dresden under the ID BO-EK-444102022.

### Experimental setup

We used the image data from the DACHS study to train a Deep Learning network to detect MSI in CRC. In this study, only information on MSI status was available, but no information on either *POLE* or *POLD1* status. The methodology we used here corresponds to that of Wagner et al. ^53^. Briefly, we tessellated all digitized whole slide images (WSIs) into tiles and stain normalized them. We further processed the normalized tiles by applying a pre-trained network to extract features from each tile. Based on these features we trained a transformer-based architecture with a classifying multilayer perceptron to classify each slide as MSI or MSS. The ground truth for all cohorts is taken from the clinical data. For TCGA the microsatellite classes contain MSI-H (high grade MSI), MSI-L (low-grade MSI) and MSS. For this study, MSI-H cases are assumed to be MSI while MSI-L and MSS cases both are assigned to MSS as performed by Wagner et al. ^53^. All experiments were performed using 5-fold cross-validation with internal validation on DACHS and external validation on two datasets. For testing the trained models from the 5-fold cross-validation, they were deployed on the external cohorts, TCGA and APHP, for which the *POLE*/*POLD1* mutation status was available. Our hypothesis applies to *POLE/POLD1* mutations that are meant to include driver mutations only while other genetic alterations like splice or passenger mutations were considered as wild type. For each slide we calculated the classification probabilities of being MSI predicted by the model. We assessed the model performance using the median AUROC of the 5 deployed models on the TCGA external cohort. To investigate the scattering of the models’ prediction results, we visualized the results of all folds for each sample respectively and highlighted the prediction score obtained from the median model. For explainability, attention heatmaps as well as prediction heatmaps of selected slides were generated. Training and deployment were performed on a NVIDIA RTX A6000 with 48 GB GPU memory. Detailed information on the Deep Learning methods (Fig. 1b) is provided in Supplementary File 1: Supplementary Methods.

### Statistics

The primary statistical endpoint for assessment of model performance during training was the AUROC including 95% confidence intervals (CIs). For internal validation (DACHS) as well as for external validation on TCGA, the CI was calculated based on all 5-fold-wise AUROCs. For calculating the statistical power of the AUROCs, we used a two-sided t-test comparing the prediction scores. For individual folds, sensitivity, specificity, positive predictive value (PPV) and negative predictive value (NPV) were calculated for each subgroup consisting of MSI, MSS and *POLE* cases respectively. For the TCGA and APHP cohorts, we calculated the mean and standard deviation of the prediction scores for each subgroup (MSI, MSS, *POLE*/*POLD1*) for the 5 folds. To do this, we first calculated the mean and standard deviation of the prediction scores for each sample over the 5 folds and then determined the mean of these values over all samples within each subgroup. In the *POLE*/*POLD1* subgroup, only driver mutations were taken into account. For hypothesis testing of differences between relative frequencies of morphologic features, a two-sided Fisher’s exact test was used.

### Reporting summary

Further information on research design is available in the Nature Research Reporting Summary linked to this article.

### Supplementary information

Supplemental material

Reporting summary

## Data Availability

The whole slide images (WSIs), molecular and clinical data for the TCGA cohort are publicly accessible at https://portal.gdc.cancer.gov/ and https://www.cbioportal.org/ (accessed 12 October 2022). For this study we used the MSI status for the TCGA cohort based on the findings of Liu et al.^57^. at https://github.com/KatherLab/cancer-metadata/blob/main/tcga/liu.xlsx (accessed 06 November 2022). The datasets from DACHS and APHP are available from the corresponding author on reasonable request. All data generated or analyzed during this study are included in this published article and its supplementary information files.

## Code availability

All source code for image processing is publicly available at https://github.com/KatherLab and given with the exact commit ID for reasons of reproducibility. The tesselation script is available at https://github.com/KatherLab/preprocessing-ng and followed by normalization with the script and the reference image available at https://github.com/KatherLab/preProcessing. Extraction of CTransPath features was carried out with scripts from https://github.com/KatherLab/marugoto as well as the transformer-based Deep Learning model and the heatmaps for explainability used for the study. Links to the exact version to the repositories including the scripts used can be found in Supplementary Table 4.
