# Supplementary material for: Deep learning for dual detection of microsatellite instability and POLE mutations in colorectal cancer histopathology
Source: NPJ Precis Oncol. 2024 May 23;8:115. doi: 10.1038/s41698-024-00592-z (PMC11116442; doi:10.1038/s41698-024-00592-z)
Supplement: Supplementary file 1 — Supplemental material [file 41698_2024_592_MOESM1_ESM.pdf]

Supplementary Information

Supplementary File 1: Supplementary Methods

Genetic testing of samples

For DACHS, the MSI status was determined with a three-plex PCR assay using the mononucleotide markers BAT25, BAT26, and CAT25 in tissue obtained from the formalin-fixed paraffin-embedded tumor block. This three-plex marker panel has a 100% concordance of MSI-H tumors compared with the National Cancer Institute/International Collaborative Group on HNPCC (NCI/ICG-HNPCC) marker panel, which includes the five markers BAT25, BAT26, D17S250, D2S123, and D5S346 [1,2]. For both APHP cohorts, all samples used had previously undergone molecular analysis in the Department of Pathology of CHU Henri Mondor between 2015 and 2023 for determination of *KRAS* and *NRAS* status, in compliance with French regulations. DNA samples had been extracted, after macrodissection when necessary, from formalin-fixed paraffin-embedded (FFPE) tissue sections using the Maxwell 16 FFPE Plus LEV DNA Purification Kit IVD (Promega, Charbonnières-les-Bains, France), according to the manufacturer's instructions. The DNA was quantified using a Qubit fluorimeter in combination with the Qubit dsDNA HS Array Kit (ThermoFisher Scientific, Waltham, MA, USA). *POLE* status was determined using High Resolution Melting (HRM) performed with a LightCycler® 480 (Roche, Basel, Switzerland) using specific primers as described previously [3]. Patients with mutated profiles identified by HRM PCR were then sequenced by Next Generation Sequencing (NGS) on S5 sequencer using the Ion AmpliSeq™ OST+ V2 panel (ThermoFisher Scientific) [3]. MSI status was determined using the fully automated Idylla™ MSI Test. IHC analysis was performed for MMR-D samples. IHC was carried out on FFPE tissue sections using antibodies against *MLH1* (mouse mAb, clone G168.728, Microm Micotech), *PMS2* (mouse mAb, clone A16-4, 1:100, BD Pharmingen), *MSH2* (mouse mAb, clone FE11, 1:100, Biocare Medical) and *MSH6* (rabbit mAb, clone EP49, 1:100, CliniSciences). Tumor mutational burden (TMB) was assessed for a subset of patients with *POLE* mutation in the APHP cohorts using FoundationOne® CDX (Roche). Tumors were considered ultra-mutated if they contained more than 100 mutations/ Megabases (mt/Mb), hypermutated if they contained between 10 and 100 mt/Mb and with low TMB if

they contained less than 10 mt/Mb [4]. For TCGA, the MSI status was retrieved from Liu et al. [5]. Briefly, they assessed MSI status with a MSI-Mono-Dinucleotide assay, that examines four mononucleotide repeat loci (BAT25, BAT26, BAT40 and transforming growth factor receptor type II) and three dinucleotide repeat loci (D2S123, D5S346 and D17S250).

Details on the Deep Learning methods

We processed the data using our open source repositories. First we tessellated the WSI into tiles of size 224 x 224 pixels which corresponds to 256 x 256 microns per slide dimension. A Canny edge detector [6] is applied to detect and remove background and blurred regions. The resulting image tiles are then normalized to reduce a potential batch effect due to different staining techniques that could degrade the model's generalizability. We used the normalization method from Macenko et al. [7] with a representative stain reference image tile taken from the DACHS cohort, which can be found in the directory used for normalization. The normalized tiles are stored on a local drive for further processing.
Similar to Wagner et al. [8] we extracted a 768-dimensional feature representation for each tile using the CTransPath feature extractor from Wang et al. [9]. CTransPath is based on an architecture consisting of a convolutional neural network (CNN) followed by a multi-scale Swin Transformer [10]. It is pre-trained in an unsupervised manner on approximately 15 million unlabeled patches tessellated from over 30 thousand WSIs derived from multiple organs and cancer types. This sample diversity ensures a certain degree of universal applicability to different cancer types and increases the generalizability of the extractor. The feature embeddings for all tiles are stacked to form a WSI level representation and stored for the subsequent Deep Learning procedure.
Feature extraction is followed by feature aggregation using a weakly supervised transformer based model for the classification task. The model takes the normalized tile embeddings of each WSI as input and predicts the probability of MSI for each WSI. The final prediction is based on the individual predictions at the tile embedding level, which are aggregated into a WSI level score [11]. More specifically, the tile embeddings for all n tiles of one WSI are stacked into an n x 768 matrix representing the WSI level embedding. In a subsequent linear projection layer followed by a ReLu activation the second dimension is reduced to 512. A class token [12] of dimension 1 x 512 is concatenated to the matrix, resulting in an (n

+ 1) x 512 matrix. The class token is randomly initialized and trained as part of the network. The matrix including the token is fed into the two 8-headed transformer layers. The input is processed in each layer with a layer normalization followed by the transformer heads and the resulting representation is computed in a second layer norm followed by a multi layer perceptron. The output of the second transformer layer is then passed to the classification layer, which consists of a layer normalization followed by a linear layer that takes the class token as input and yields a final binary prediction for MSI.

The model is trained using the AdamW optimizer [13] with a learning rate of 2e-5 and a weight decay of 2e-5 according to Wagner et al. [8]. To address the imbalance in MSI and MSS cases, a weighted cross-entropy loss function is applied, which assigns higher weights to underrepresented classes, thus ensuring balanced consideration of both classes during model training. Our model is trained for 32 epochs with a batch size of 64 that corresponds to the number of WSIs that make up one iteration of an epoch. This is in contrast to Wagner et al. who used a batch size of one and trained the model for eight epochs. The main reason for this is that we randomly sample a bag of maximum 512 tiles per batch. If there are less than 512 tiles the remainder is filled with zeros so that we get the same shape for each bag. The bag is resampled each epoch. During training the predictive performance of the model is validated by using all tiles from each WSI in the validation dataset. We did the same for model deployment on the external datasets. For selected slides, attention maps were calculated and visualized so that a visual alignment of the attention map with the original slides is possible for explainability reasons. Regions of high attention mean high influence of this region to the algorithm's predictions and are highlighted in yellow/green while regions of low attention are colored blue. Due to the transformer’s architecture, which consists of 2 layers and 8 heads, 16 heatmaps are generated for each slide.

Supplementary File 2: Supplementary Figures and Tables

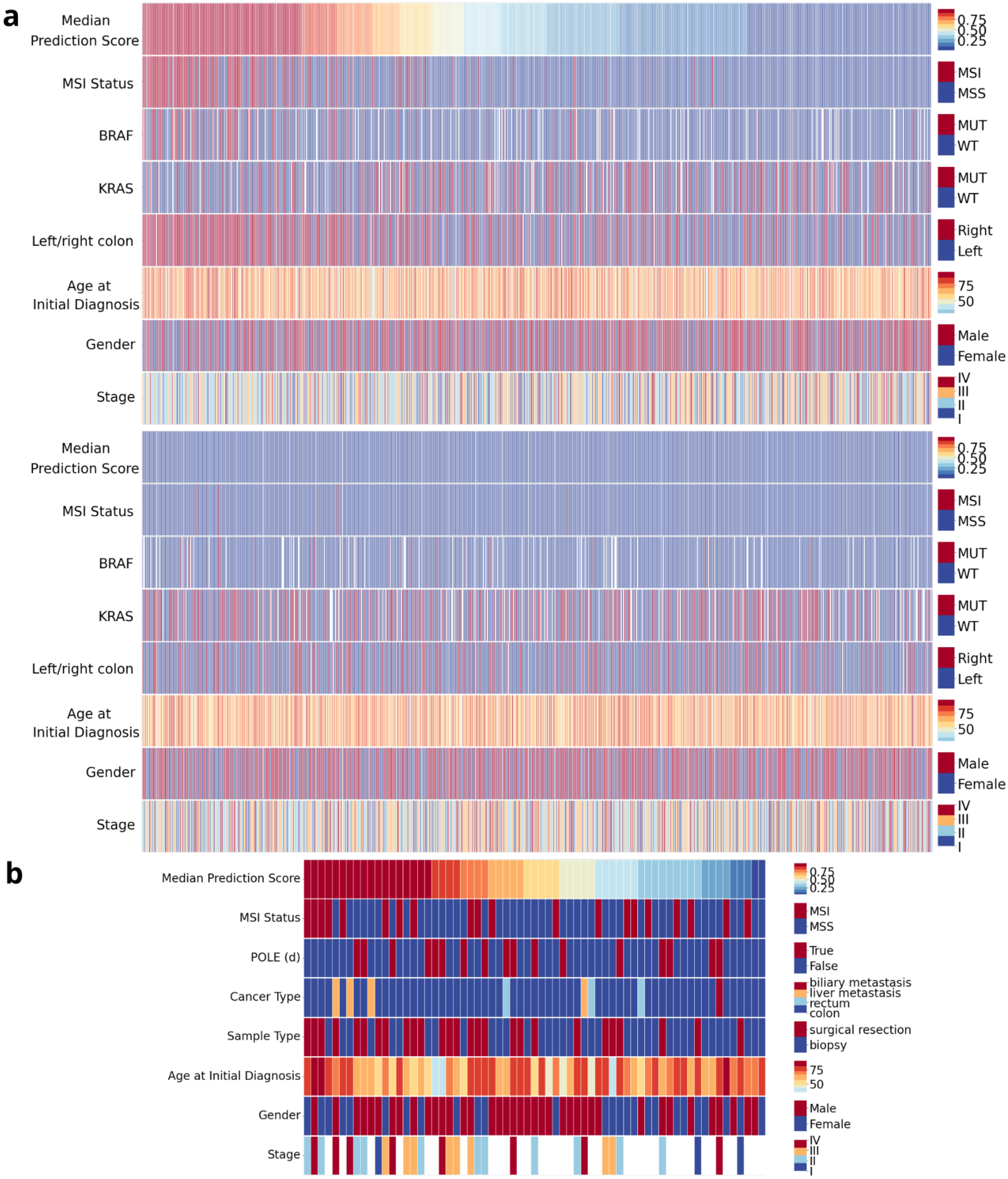

Supplementary Figure 1: Patient and molecular characteristics for the cohorts used in this study. a Molecular characteristics for the DACHS (Darmkrebs: Chancen der Verhütung durch Screening) cohort. The columns correspond to individual patients and are sorted by "Median Prediction Score",

representing the median model of five-fold cross-validation evaluated using AUROC. In order to visualize the large amount of samples, the chart has been split in half and stacked on each other. "MSI Status" is the ground truth of microsatellite status. TMB represents tumor mutational burden. For BRAF and KRAS “MUT” indicates the presence of the respective mutation and “WT” the absence of the mutation (wild type). “Stage” ranges from one to four and indicates the cancer stage. The same range is used by “Colorectal CMS” which stands for the consensus molecular subtypes (CMS) of colorectal cancer. Empty cells represent cases where the information regarding specific characteristics is missing. **b** Molecular characteristics for the APHP (Assistance Publique–Hôpitaux de Paris) cohort. The columns correspond to individual patients and are sorted by "Median Prediction Score", representing the median model of five-fold cross-validation evaluated using AUROC. "MSI Status" is the ground truth of microsatellite status. *POLE* (d) denotes patients with a *POLE* driver mutation. “Stage” ranges from one to four and indicates the cancer stage. Empty cells represent cases where the information regarding specific characteristics is missing.

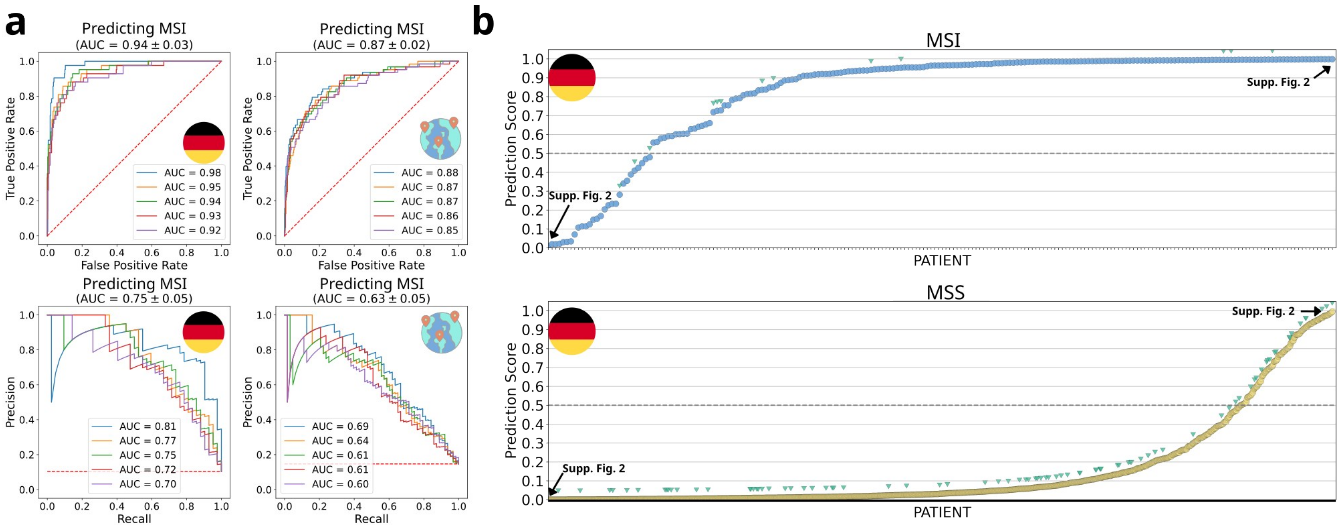

Supplementary Figure 2: Results of MSI prediction experiments with Vision Transformer based pipeline. Results are shown for training on DACHS (Darmkrebs: Chancen der Verhütung durch Screening, Germany) and testing on TCGA (The Cancer Genome Atlas, International). **a** Receiver Operating Characteristics Curve (ROC) and Precision-Recall Curve (PRC) for training on DACHS (left) and testing on TCGA (right). Each line represents one fold from five-fold cross validation. The mean area under the curve (AUROC) is calculated for each experiment with standard deviation. MSI compared to MSS in TCGA gives an AUROC of 0.87±0.02 (top right ROC). When positive cases are defined as either MSI or POLE driver mutations, and all others as negative, the AUROC is 0.86±0.01 (not shown). The ROC and PRC were not calculated for APHP (Assistance Publique–Hôpitaux de Paris) due to low samples and high relative *POLE* cases that are hypothesized to be classified as MSI. **b** In the DACHS training cohort, prediction scores were calculated for patients with MSI (top chart) and MSS/MSI-L (bottom chart). Each patient is represented by a dot and predictions for all patients are gathered from the test set in each of the five folds. Early-onset colorectal cancer patients (age at diagnosis <50 years) [14] are indicated by a green triangle. Arrows point to the corresponding heatmaps of selected samples. The icons indicating the origin of the cohorts are sourced from www.flaticon.com.

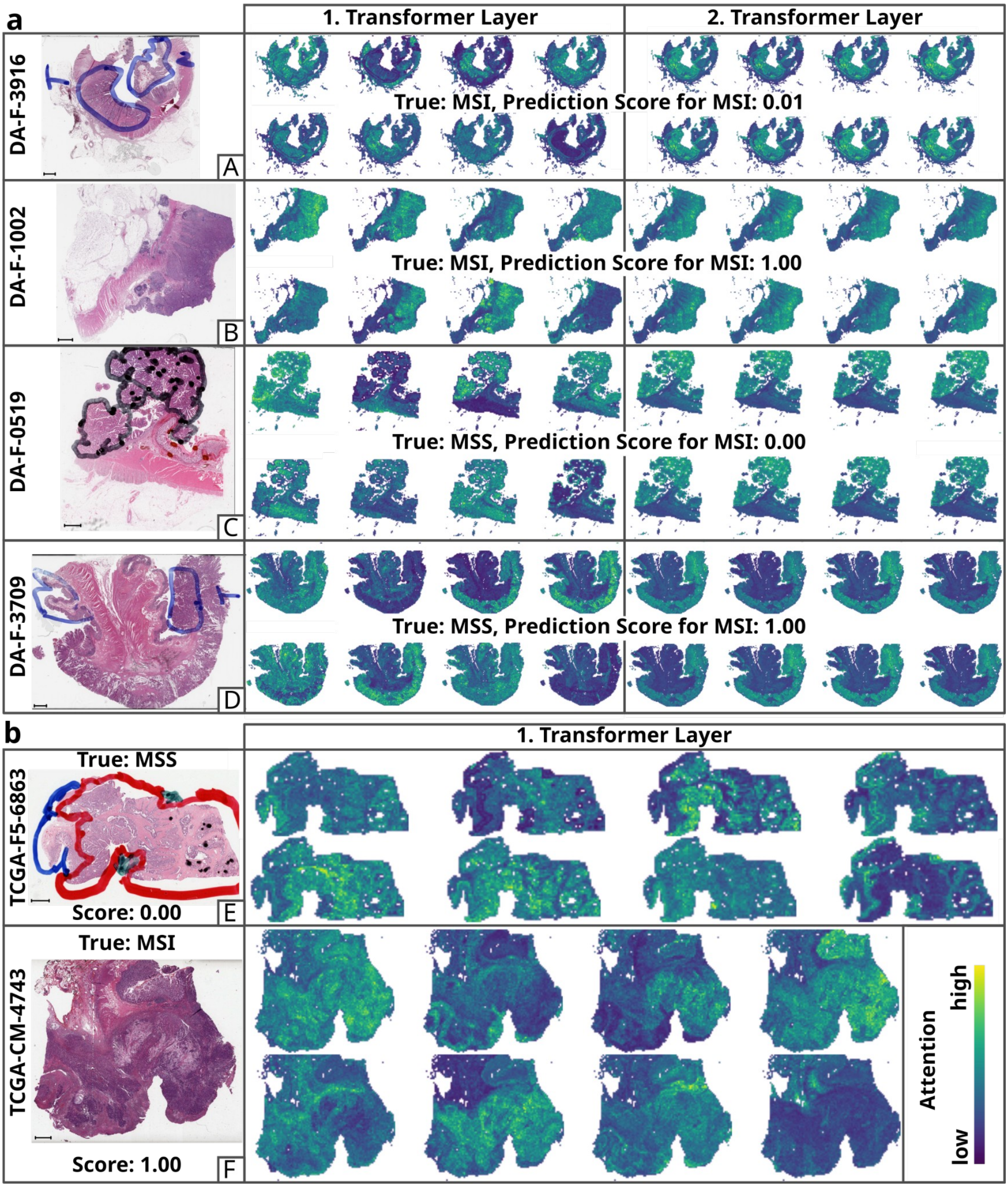

Supplementary Figure 3: Attention heatmaps for selected samples. The heatmaps were created for the model with median AUROC of the five trained models deployed on the TCGA (The Cancer Genome Atlas) cohort. Due to the transformer's architecture, which consists of 2 layers and 8 heads, 16 heatmaps are generated for each slide. Blue regions indicate low and yellow regions high attention and

thus high relevance of the respective region for the prediction. The scale bars indicate a length of 2 mm. **a** Heatmaps for selected slides from DACHS (Darmkrebs: Chancen der Verhütung durch Screening) with ground truth microsatellite status (“True”) and prediction score for MSI. Cases with low MSI predictions scores display conventional gland-forming morphology (A: DA-F-3916; C: DA-F-0519), whereas cases with a high MSI prediction score shows a medullary growth pattern with high numbers of tumor-infiltrating lymphocytes (B: DA-F-1002) or a mucinous differentiation (D: DA-F-3709). **b** Heatmaps for the first transformer layer for selected slides from the TCGA cohort. In TCGA, cases with low MSI predictions scores again show a conventional gland-forming morphology (E: TCGA-F5-6863), whereas high MSI predictions scores are linked to a mucinous differentiation (F: TCGA-CM-4743).

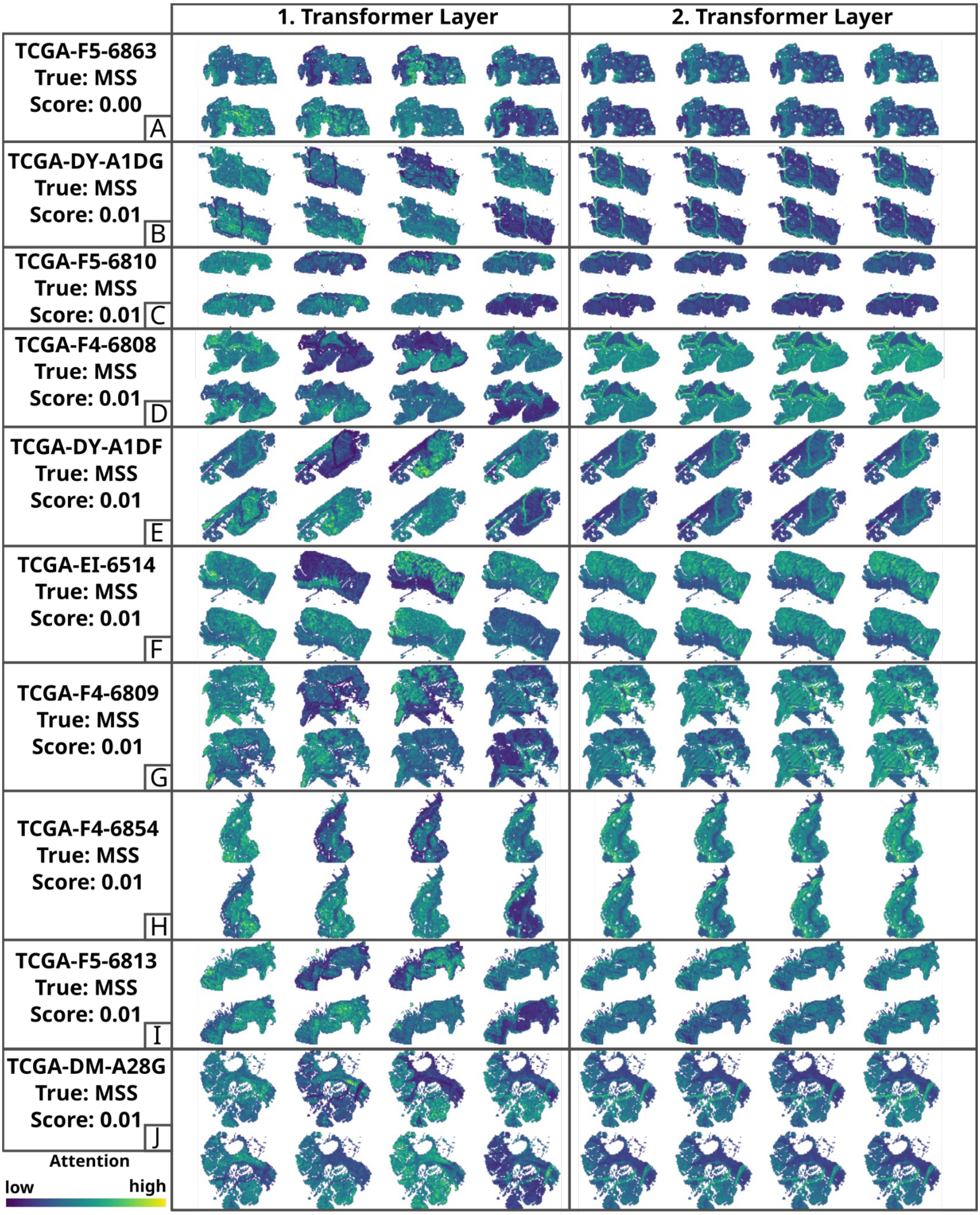

Supplementary Figure 4: Attention heatmaps for samples with lowest prediction scores from the TCGA (The Cancer Genome Atlas) cohort. The heatmaps were created for the model with median AUROC of the five trained models deployed on TCGA. Due to the transformer's architecture, which

consists of 2 layers and 8 heads, 16 heatmaps are generated for each slide. Blue regions indicate low and yellow regions high attention and thus high relevance of the respective region for the prediction. The associated whole slide images can be viewed under the GDC Data Portal: https://portal.gdc.cancer.gov/. Results from pathological examination of the images can be found in Supplementary Table 5.

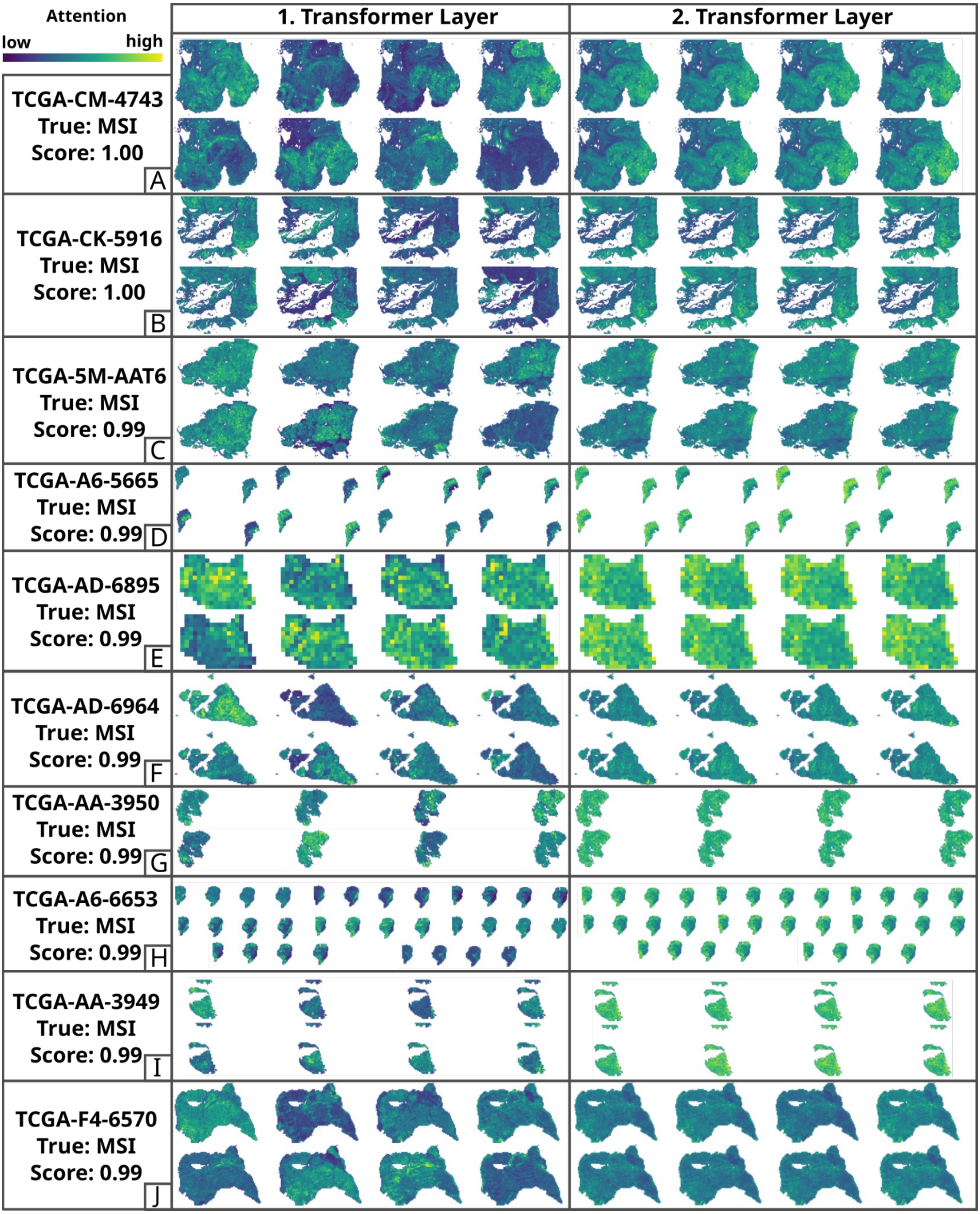

Supplementary Figure 5: Attention heatmaps for samples with highest prediction scores from the TCGA (The Cancer Genome Atlas) cohort. The heatmaps were created for the model with median AUROC of the five trained models deployed on TCGA. Due to the transformer's architecture,

which consists of 2 layers and 8 heads, 16 heatmaps are generated for each slide. Blue regions indicate low and yellow regions high attention and thus high relevance of the respective region for the prediction. The associated whole slide images can be viewed under the GDC Data Portal: https://portal.gdc.cancer.gov/. Results from pathological examination of the images can be found in Supplementary Table 5.

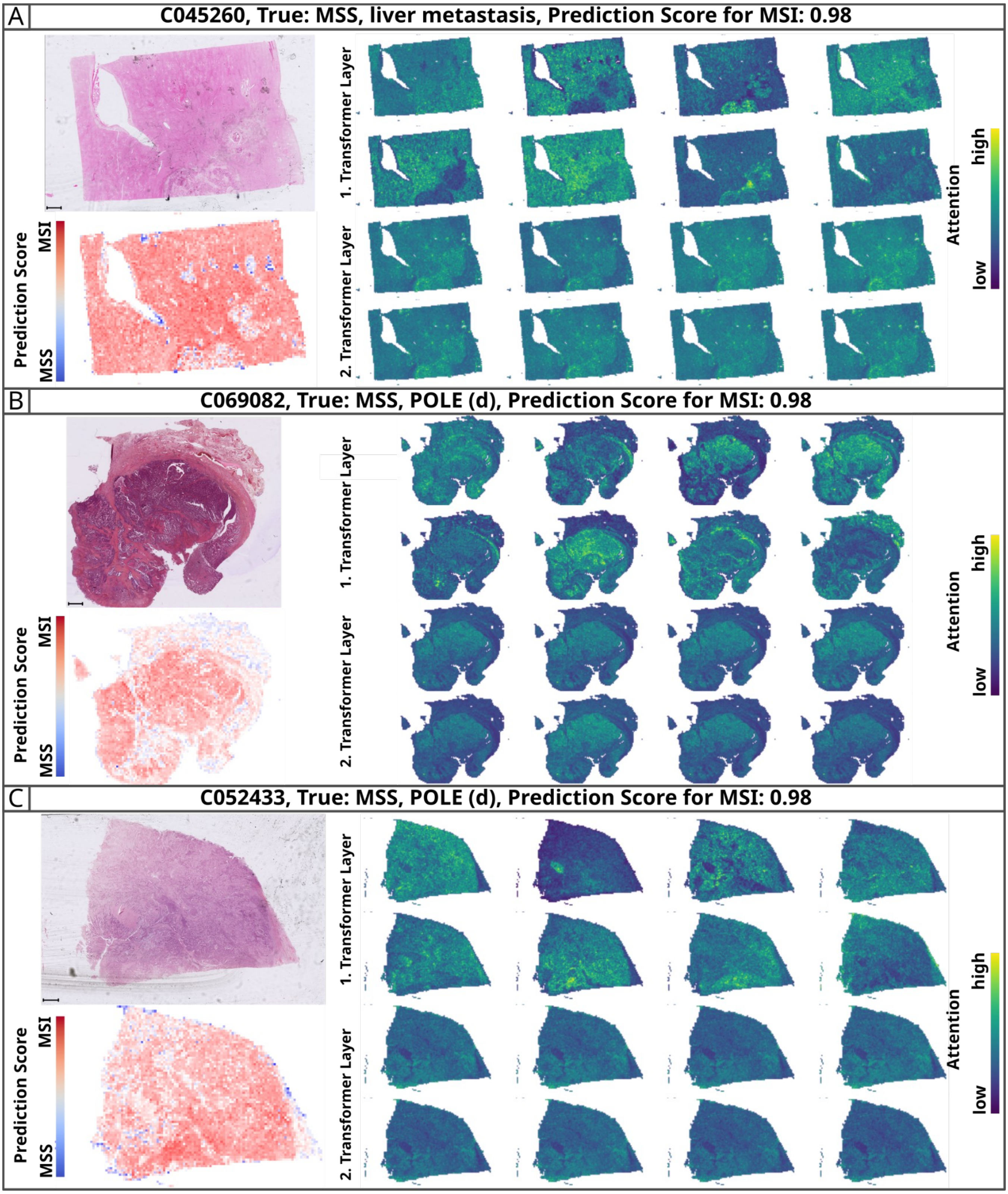

Supplementary Figure 6: Prediction and attention heatmaps for selected samples with highest prediction scores from the APHP (Assistance Publique–Hôpitaux de Paris) Resection cohort. The heatmaps were created for the model with median AUROC of the five trained models deployed on the TCGA (The Cancer Genome Atlas) cohort. The prediction heatmap (A-C, bottom left) highlights the

tiles classified as MSI (red) and MSS (blue). The color intensity indicates the amount of attention paid to the respective tile with high attention corresponding to high intensity. Due to the transformer's architecture, which consists of 2 layers and 8 heads, 16 attention heatmaps are generated for each slide (A-C, right). Dark blue regions indicate low and yellow regions high attention and thus high relevance of the respective region for the prediction. The scale bars indicate a length of 2 mm.

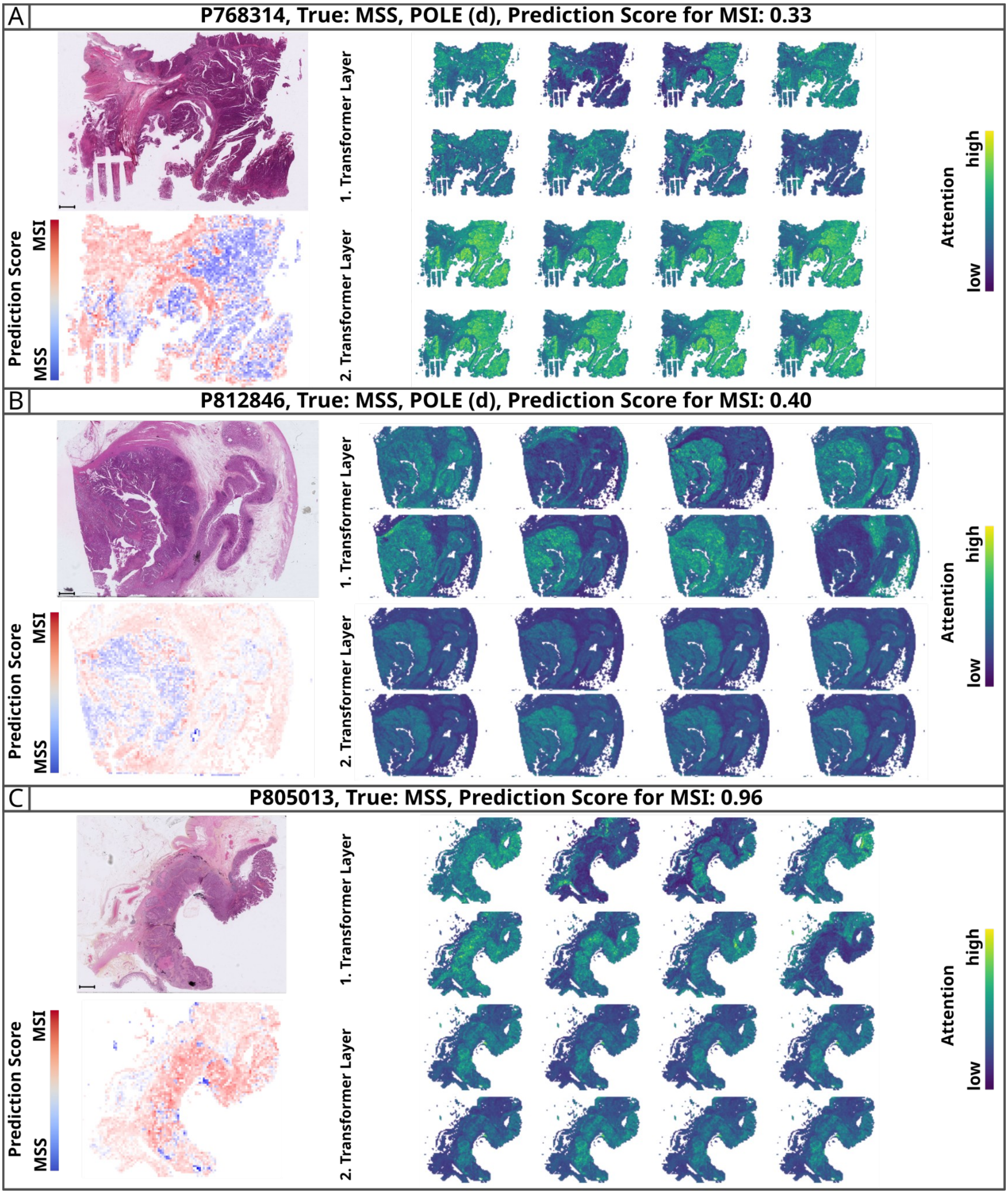

Supplementary Figure 7: Prediction and attention heatmaps for selected samples from the APHP (Assistance Publique–Hôpitaux de Paris) Resection cohort. The heatmaps were created for the model with median AUROC of the five trained models deployed on the TCGA (The Cancer Genome Atlas) cohort. The prediction heatmap (A-C, bottom left) highlights the tiles classified as MSI (red) and

MSS (blue). The color intensity indicates the amount of attention paid to the respective tile with high attention corresponding to high intensity. Due to the transformer's architecture, which consists of 2 layers and 8 heads, 16 attention heatmaps are generated for each slide (A-C, right). Dark blue regions indicate low and yellow regions high attention and thus high relevance of the respective region for the prediction. The scale bars indicate a length of 2 mm.

Supplementary Table 1: Sociodemographic and clinicopathological patient characteristics of the DACHS (Darmkrebs: Chancen der Verhütung durch Screening), TCGA (The Cancer Genome Atlas) and APHP (Assistance Publique–Hôpitaux de Paris) cohorts.

Parameter	Description	Cohort		
		DACHS	TCGA	APHP
**Use in study**		train	test	test
**N (Clinical data)**	Patients with clinical data	2448	926	66
**N (Slides)**		3618	625	66
**N (Features)**		3617	589	65
**N (Processed)**	Patients included in study	2039	429	65
**Age**	Median	70	67.1	-
	Interquartile range	15	18.1	-
	Patients younger than 50 years (Early-Onset Colorectal Cancer)	100 (4.9%)	58 (13.5%)	-
**Gender**	Male	1180 (57.9%)	212 (49.4%)	-
	Female	859 (42.1%)	214 (49.9%)	-
	Unknown	0 (0.0%)	3 (0.7%)	65 (100%)
**Tumor Stage**	Stage 1	375 (18.4%)	71 (16.6%)	-
	Stage 2	684 (33.5%)	150 (35.0%)	-
	Stage 3	695 (34.1%)	134 (31.2%)	-
	Stage 4	285 (14.0%)	58 (13.5%)	-
	Stage unknown	0 (0.0%)	16 (3.7%)	65 (100%)
**Tumor Location**	Right Colon	717 (35.2%)	168 (39.2%)	-
	Left Colon	1307 (64.1%)	227 (52.9%)	-
	Unknown	15 (0.7%)	34 (7.9%)	65 (100%)
**MSI Status**	MSI-H	210 (10.3%)	63 (14.7%)	20 (30.8%)
	MSI-L/MSS	1829 (89.7%)	366 (85.3%)	45 (69.2%)
**KRAS Status**	Wild type	1281 (62.8%)	241 (56.2%)	-

Parameter	Description	Cohort		
		DACHS	TCGA	APHP
	Mutated	610 (29.9%)	188 (43.8%)	-
	Unknown	148 (7.3%)	0 (0.0%)	65 (100%)
**BRAF Status**	Wild type	1729 (84.8%)	380 (88.6%)	-
	Mutated	141 (6.9%)	49 (11.4%)	-
	Unknown	169 (8.3%)	0 (0.0%)	65 (100%)
**Race**	White	-	243 (56.6%)	-
	Black/African American	-	57 (13.3%)	-
	Asian	-	12 (2.8%)	-
	American Indian/Alaska Native	-	1 (0.2%)	-
	Unknown	-	116 (27.0%)	65 (100%)
**Neoadjuvant Therapy Prior To Resection**	No	1822 (89.4%)	428 (99.8%)	-
	Yes	211 (10.3%)	0 (0.0%)	-
	Unknown	6 (0.3%)	1 (0.2%)	65 (100%)

Supplementary Table 2: Molecular characteristics and prediction scores of cases with *POLE* and *POLD1* mutations from the TCGA (The Cancer Genome Atlas) cohort. Driver mutations are marked with a "(d)" [15,16]. Gender is represented by 'F' for Female and 'M' for Male. When possible, the TNM stage and tumor grade (enclosed in brackets) are provided for tumors. The table is arranged in descending order, starting with the highest prediction score at the top, obtained by the model with the median AUROC of the five trained models deployed on TCGA. The exonuclease domain of *POLE* is located between amino acid residues 268 and 471 [17].

**PATIENT**	**Age**	**Gender**	**Stage (Grade)**	**MSI Status**	***POLE* Protein Change**	***POLD1* Protein Change**	**Prediction Score**
TCGA-CK-5916	71.25	F	T1N0M0 (I)	MSI		S816del	1.00
TCGA-CM-4743	69.24	M	T3N0M0 (IIA)	MSI		X987_splice	1.00
TCGA-5M-AAT6	40.66	F	T4aN2bM1a (IV)	MSI	T1313M	V765M	0.99
TCGA-A6-3809				MSI	D1214A	R990H	0.99
TCGA-A6-5661	80.62	F	T3N0M0 (IIA)	MSI		G395D	0.99
TCGA-A6-5665	84.64	F	T3N0M0 (IIA)	MSI		V933Cfs*21	0.99
TCGA-AA-3949	87.83	F	T3N1M0 (IIIB)	MSI		Q461H	0.99
TCGA-AD-6964	58.75	M		MSI	Y473C	X669_splice	0.99
TCGA-CA-6718	46.84	M	T3N0M0 (IIA)	MSS	P286R (d)		0.99
TCGA-CK-4951	79.35	F	T3N0M0 (IIA)	MSI	E2137K	A215V, Y607H	0.99
TCGA-CK-6751	88.58	F	T2N0MX (I)	MSS		E1040G	0.99
TCGA-F4-6570	78.77	F	T3N0M0 (IIA)	MSI	E396Sfs*16	R1008C	0.99
TCGA-G4-6588	58.95	F	T3N0M0 (IIA)	MSI	A2030Pfs*18	R195Q	0.99
TCGA-AA-3811	84.58	F	T3N2M0 (III)	MSI	Y1003C		0.98
TCGA-AA-3947	60.33	F	T4N0M0 (IIB)	MSI		P787L	0.98
TCGA-AG-3892	57.17	F	T1N0M0 (I)	MSS	S459F (d)		0.98
TCGA-CK-5913	58.59	F	T3N0MX (IIA)	MSI		V596I	0.98
TCGA-CM-5861	63.66	F	T3N0M0 (IIA)	MSI	V2152M	X987_splice	0.98

PATIENT	Age	Gender	Stage (Grade)	MSI Status	*POLE* Protein Change	*POLD1* Protein Change	Prediction Score
TCGA-D5-6927	34.87	M	T3N0M0 (IIA)	MSI		L427I	0.98
TCGA-EI-6507	60.72	M	T3N0M0 (IIA)	MSI		L852R	0.98
TCGA-EI-6917	33.16	M	T3N1M0 (IIIA)	MSS	V411L (d), A426V	L1044P	0.98
TCGA-WS-AB45	52	F	T3N0MX (IIA)	MSI	A2030Pfs*18, V2152M, K1383R, R924H	R253Q	0.98
TCGA-AA-A010	46.33	F	T4N0M0 (IIB)	MSI-L	P436R (d), A189T		0.97
TCGA-AD-5900	67.83	M	T2N0MX (I)	MSI		T441M	0.97
TCGA-AY-6197	60.12	M		MSI		A864T, C873R	0.97
TCGA-D5-6540	66.48	M	T2N0M0 (I)	MSI	A1224T		0.97
TCGA-NH-A50U	42.3	M	T4aN0M1a (IVA)	MSS	A2040V		0.96
TCGA-A6-2686	81.1	F	T3N0M0 (IIA)	MSI	K1170Nfs*49	R180Gfs*3	0.95
TCGA-AZ-6598	77.16	F	T3N0MX (II)	MSI	X1517_splice, T1052M	P116Hfs*53	0.95
TCGA-CA-6717	57.45	M	T3N0M0 (IIA)	MSS	L1235I, R1371*		0.95
TCGA-A6-4105	79.66	M	T3N0M0 (IIA)	MSS		X380_splice	0.94
TCGA-F5-6814	57.03	M	T2N0M0 (IIA)	MSS	P286R (d)		0.93
TCGA-CM-6162	48.25	F	T3N1aM0 (IIIB)	MSI	D2166N	R683H	0.91
TCGA-D5-6930	67.8	M	T3N0M0 (IIA)	MSI		P116Hfs*53	0.90
TCGA-AA-3833	63	F	T3N0M0 (IIA)	MSI	R1160H		0.84
TCGA-AZ-4315	61.16	M	T3N0M0 (IIA)	MSS	V411L (d), R1826W,		0.83

PATIENT	Age	Gender	Stage (Grade)	MSI Status	*POLE* Protein Change	*POLD1* Protein Change	Prediction Score
					R1556W		
TCGA-AA-3864	71.83	M	T3N0M0 (II)	MSI	R231H		0.82
TCGA-NH-A5IV	90	F	T3N0MX (IIA)	MSI	L698Cfs*94	R454C, R225H	0.74
TCGA-EI-6882	59.36	M	T3N0M0 (IIA)	MSI	R759C		0.72
TCGA-F5-6464	77.12	F	T4bN2aM0 (IIIC)	MSS		I878V	0.64
TCGA-AZ-4615	84.62	M	T3N1M0 (IIIB)	MSI	K325Sfs*19		0.59
TCGA-AA-3984	61.84	F	T3N0M0 (IIA)	MSS	V411L (d)		0.57
TCGA-AA-3977				MSS	F367S (d), K777N		0.53
TCGA-AA-3678	60.83	F	T2N1M0 (III)	MSS	D1752N		0.37
TCGA-AA-A00N				MSI	V411L (d), L1255V		0.31
TCGA-AG-A002	35.84	M	T2N0M0 (I)	MSS	S459F (d), R150*		0.12
TCGA-AG-A01W	67.75	F	T3N0M0 (II)	MSS	D2013N		0.02
TCGA-CM-6678	63.16	F	T4aN1cM1a (IVA)	MSS	G628R		0.01
TCGA-F4-6806	59.21	F	T2N0M0 (I)	MSS	K778del		0.01

Supplementary Table 3: Tissue sample specifications, molecular characteristics and prediction scores of cases with *POLE* mutations from the APHP (Assistance Publique–Hôpitaux de Paris) cohort. "TMB" indicates the tumor mutational burden which was assessed only for a small proportion of samples in this cohort. The cutoffs are set to <10 mutations/Mb for low TMB, between 10-100 mutations/Mb for medium TMB and >100 mutations/Mb for high TMB. Driver mutations are marked with a "(d)". Gender is represented by 'F' for Female and 'M' for Male. When possible, the TNM stage and tumor grade (enclosed in brackets) are provided for tumors. The table is arranged in descending order, starting with the highest prediction score at the top, obtained by the model with the median AUROC of the five trained models deployed on TCGA. The exonuclease domain of POLE is located between amino acid residues 268 and 471 [17].

Patient	Cancer Type	Sample Type	Age	Gender	Stage (Grade)	MSI Status	*POLE* Protein Change	TMB	Prediction Score
C045260	liver metastasis	surgical resection	71	M	(IV)	MSS	L425R	low TMB	0.98
C069082	colon	surgical resection	66	M	pT3N0 (II)	MSS	V411L (d)	unknown	0.98
C052433	colon	surgical resection	63	M	pT4N0 (II)	MSS	P286R (d)	high TMB	0.97
C067109	colon	biopsy	53	M	(IV)	MSS	S459F (d)	unknown	0.95
C067647	colon	surgical resection	66	F	pT4N2a (III)	MSS	V474I	unknown	0.94
P733928	colon	surgical resection	57	M	pT4aN2b (III)	MSI	V464V	unknown	0.93
C054568	colon	biopsy	36	M		MSS	V411L (d)	high TMB	0.90
P782249	colon	biopsy	52	M		MSS	P286R (d)	unknown	0.90
C053306	colon	surgical resection	42	M	pT4N0M1 (IV)	MSS	P286R (d)	high TMB	0.89

Patient	Cancer Type	Sample Type	Age	Gender	Stage (Grade)	MSI Status	*POLE* Protein Change	TMB	Prediction Score
C054128	colon	biopsy	55	M		MSS	P286R (d)	high TMB	0.80
C043418	colon	surgical resection	73	F	pT3N0 (II)	MSS	V411L (d)	high TMB	0.74
C061434	colon	surgical resection	76	M	pT3N0M1 (IV)	MSS	S459F (d)	unknown	0.69
C068867	rectum	biopsy	65	M		MSS	P286R (d)	unknown	0.69
P787324	colon	biopsy	79	M		MSS	M299V	unknown	0.62
C061043	colon	surgical resection	55	M	pT3N0 (II)	MSS	P286R (d)	unknown	0.61
C072096	colon	biopsy	74	M		MSS	A456P	unknown	0.60
C070325	colon	biopsy	44	M		MSS	A456P	unknown	0.58
C055606	colon	biopsy	47	M		MSS	P286R (d)	medium TMB	0.52
C051634	rectum	biopsy	47	M		MSS	S461T	high TMB	0.46
P812846	colon	surgical resection	76	F	pT3N0 (II)	MSS	S459F (d)	unknown	0.40
C068546	rectum	biopsy	57	M		MSS	D275A	unknown	0.36
P812504	colon	biopsy	75	F	pT2	MSS	D287E	unknown	0.35
P768314	colon	surgical resection	61	M	pT3N0 (II)	MSS	P286R (d)	unknown	0.33
P786445	colon	biopsy	68	M		MSS	V411L (d)	unknown	0.32
C055951	colon	biopsy	61	M		MSS	S459F (d)	medium TMB	0.22
P776044	liver metastasis	biopsy	79	M	(IV)	MSS	P286R (d)	unknown	0.22
C053509	colon	biopsy	79	F		MSS	V406A	low TMB	0.04

Supplementary Table 4: Code availability. Links to exact versions of the directories containing the code used for the study as mentioned in the methods section.

Process	Link
Tessellation	https://github.com/KatherLab/preprocessing-ng/tree/1f5fdebf669363cf67bb422b-b4cb0f91218d9c29
Normalization	https://github.com/KatherLab/preProcessing/tree/505b3b4986f2c73cf9e1412ccf-ba7de047bcb62c
Feature extraction with CTransPath	https://github.com/KatherLab/marugoto/tree/d401e1157635273cd4a99ca6e60c83d-b7ea09a22
Transformer based Deep Learning model	https://github.com/KatherLab/marugoto/tree/c77f5331a29bd70e055e0766abb-b1c378f20b475
Heatmaps	https://github.com/KatherLab/marugoto/tree/fa627713c04e8be95bab0ce3c4280f-b497caf95b

Supplementary Table 5: Morphological features of TCGA (The Cancer Genome Atlas) cases with highest (N=10) and lowest (N=10) MSI prediction scores. The prediction scores are obtained by the model with the median AUROC of the five trained models deployed on TCGA. Pathological assessment with regards to histology (NOS: not otherwise specified versus mucinous/focally mucinous versus medullary/medullary-like), TILs (Tumor-infiltrating lymphocytes; high versus low) and presence of dirty necrosis (absent versus present) for patient slides with ten of the highest and lowest prediction scores.

PATIENT	MSI Status	MSI Prediction Score	Histology	TILs	Dirty Necrosis
TCGA-F5-6863	MSS	0.00	NOS	low	absent
TCGA-DY-A1DG	MSS	0.01	NOS	low	present
TCGA-F5-6810	MSS	0.01	NOS	low	present
TCGA-F4-6808	MSS	0.01	NOS	low	present
TCGA-DY-A1DF	MSS	0.01	NOS	low	present
TCGA-EI-6514	MSS	0.01	NOS	low	present
TCGA-F4-6809	MSS	0.01	mucinous/focally mucinous	low	present
TCGA-F4-6854	MSS	0.01	NOS	low	present
TCGA-F5-6813	MSS	0.01	NOS	low	absent
TCGA-DM-A28G	MSS	0.01	mucinous/focally mucinous	low	absent
TCGA-CM-4743	MSI	1.00	mucinous/focally mucinous	high	absent
TCGA-CK-5916	MSI	1.00	medullary/medullary-like	high	absent
TCGA-5M-AAT6	MSI	0.99	medullary/medullary-like	high	absent
TCGA-A6-5665	MSI	0.99	mucinous/focally mucinous	low	absent
TCGA-AD-6895	MSI	0.99	medullary/medullary-like	high	absent
TCGA-AD-6964	MSI	0.99	medullary/medullary-like	high	absent
TCGA-AA-3950	MSI	0.99	mucinous/focally mucinous	low	absent
TCGA-A6-6653	MSI	0.99	mucinous/focally mucinous	low	absent
TCGA-AA-3949	MSI	0.99	medullary/medullary-like	high	absent
TCGA-F4-6570	MSI	0.99	medullary/medullary-like	high	absent

Supplementary Table 6: Pathological assessment of top (highest prediction scores) and bottom (lowest prediction scores) predicted TCGA (The Cancer Genome Atlas) cases. For the differences between relative frequencies of each morphological feature from Supplementary Table 5 the statistical significance is determined with Fisher's exact test.

		**Top (N=10, highest scores)**	**Bottom (N=10, lowest scores)**	
Morphologic Feature		*n*	*n*	*p-value*
Histology				
	NOS	0	8	<0.001
	focally mucinous/mucinous	4	2	
	medullary/medullary-like	6	0	
TILs				
	low	3	10	0.002
	high	7	0	
Dirty necrosis				
	absent	10	3	0.002
	present	0	7	

Supplementary Table 7: Histopathologic review of MSS and *POLE*/*POLD1* wild type misclassified cases (N=10) with highest MSI-prediction scores from the TCGA (The Cancer Genome Atlas) cohort. The prediction scores are obtained by the model with the median AUROC of the five trained models deployed on TCGA. The associated whole slide images can be viewed under the GDC Data Portal: https://portal.gdc.cancer.gov/.

PATIENT	MSI Prediction Score	Histology
**TCGA-AG-3881**	0.99	mucinous adenocarcinoma (~50% extracellular mucin content)
**TCGA-AA-3684**	0.98	mucinous adenocarcinoma (~80% extracellular mucin content)
**TCGA-AM-5820**	0.98	adenocarcinoma NOS ('Not Otherwise Specified', gland-forming, but partly solid/low stroma content, partly medullary growth pattern)
**TCGA-D5-7000**	0.98	with mucinous differentiation (~40% extracellular mucin content)
**TCGA-AA-3858**	0.97	adenocarcinoma NOS (gland-forming, partly cribriform, low stroma content), occasionally TILs ('Tumor-Infiltrating Lymphocytes')
**TCGA-D5-6931**	0.97	adenocarcinoma NOS (gland-forming, partly cribriform, low stroma content)
**TCGA-AA-3979**	0.96	adenocarcinoma NOS (gland-forming, partly cribriform, low stroma content)
**TCGA-AG-3883**	0.96	adenocarcinoma NOS (gland-forming, partly cribriform, low stroma content), many TILs
**TCGA-AG-3894**	0.96	adenocarcinoma NOS (gland-forming, partly cribriform, low stroma content), many TILs
**TCGA-CK-4950**	0.96	mucinous adenocarcinoma (~80% extracellular mucin content)

Supplementary References

1. Boland, C. R. *et al.* A National Cancer Institute Workshop on Microsatellite Instability for cancer detection and familial predisposition: development of international criteria for the determination of microsatellite instability in colorectal cancer. *Cancer Res.* **58**, 5248–5257 (1998).
2. Findeisen, P. *et al.* T25 repeat in the 3' untranslated region of the CASP2 gene: a sensitive and specific marker for microsatellite instability in colorectal cancer. *Cancer Res.* **65**, 8072–8078 (2005).
3. Favre, L. *et al.* High prevalence of unusual KRAS, NRAS, and BRAF mutations in POLE-hypermutated colorectal cancers. *Mol. Oncol.* **16**, 3055–3065 (2022).
4. Sha, D. *et al.* Tumor Mutational Burden as a Predictive Biomarker in Solid Tumors. *Cancer Discov.* **10**, 1808–1825 (2020).
5. Liu, Y. *et al.* Comparative Molecular Analysis of Gastrointestinal Adenocarcinomas. *Cancer Cell* **33**, 721–735.e8 (2018).
6. Canny, J. A computational approach to edge detection. *IEEE Trans. Pattern Anal. Mach. Intell.* **8**, 679–698 (1986).
7. Macenko, M. *et al.* A method for normalizing histology slides for quantitative analysis. in *2009 IEEE International Symposium on Biomedical Imaging: From Nano to Macro* (IEEE, 2009). doi:10.1109/isbi.2009.5193250.
8. Wagner, S. J. *et al.* Transformer-based biomarker prediction from colorectal cancer histology: A large-scale multicentric study. *Cancer Cell* **41**, 1650–1661.e4 (2023).
9. Wang, X. *et al.* Transformer-based unsupervised contrastive learning for histopathological image classification. *Med. Image Anal.* **81**, 102559 (2022).
10. Liu, Z. *et al.* Swin Transformer: Hierarchical Vision Transformer using Shifted Windows. *arXiv:2103.14030 [cs.CV]* (2021).
11. Bilal, M. *et al.* An aggregation of aggregation methods in computational pathology. *Med. Image Anal.* **88**, 102885 (2023).
12. Dosovitskiy, A. *et al.* An Image is Worth 16x16 Words: Transformers for Image Recognition at

Scale. *arXiv:2010:11929 [cs.CV]* (2020).

13. Loshchilov, I. & Hutter, F. Decoupled Weight Decay Regularization. *arXiv:1711.05101* (2017).
14. Brockway-Lunardi, L. *et al.* Early-onset colorectal cancer research: gaps and opportunities. *Colorectal Cancer* **9**, CRC34 (2020).
15. Kather, J. N., Halama, N. & Jaeger, D. Genomics and emerging biomarkers for immunotherapy of colorectal cancer. *Semin. Cancer Biol.* **52**, 189–197 (2018).
16. Hwang, H. S., Kim, D. & Choi, J. Distinct mutational profile and immune microenvironment in microsatellite-unstable and POLE-mutated tumors. *J Immunother Cancer* **9**, (2021).
17. Church, D. N. *et al.* DNA polymerase ε and δ exonuclease domain mutations in endometrial cancer. *Hum. Mol. Genet.* **22**, 2820–2828 (2013).
