# Supplementary material for: Deep learning for dual detection of microsatellite instability and POLE mutations in colorectal cancer histopathology
Source: NPJ Precis Oncol. 2024 May 23;8:115. doi: 10.1038/s41698-024-00592-z (PMC11116442; doi:10.1038/s41698-024-00592-z)
Supplement: Supplementary file 2 — Reporting summary [file 41698_2024_592_MOESM2_ESM.pdf]

nature portfolio

Corresponding author(s): Jakob Nikolas Kather

Last updated by author(s): Feb 12, 2024

Reporting Summary

Nature Portfolio wishes to improve the reproducibility of the work that we publish. This form provides structure for consistency and transparency in reporting. For further information on Nature Portfolio policies, see our Editorial Policies and the Editorial Policy Checklist.

Statistics

For all statistical analyses, confirm that the following items are present in the figure legend, table legend, main text, or Methods section.

n/a	Confirmed	
☐	☒	The exact sample size (n) for each experimental group/condition, given as a discrete number and unit of measurement
☐	☒	A statement on whether measurements were taken from distinct samples or whether the same sample was measured repeatedly
☐	☒	The statistical test(s) used AND whether they are one- or two-sided<br>*Only common tests should be described solely by name; describe more complex techniques in the Methods section.*
☐	☒	A description of all covariates tested
☐	☒	A description of any assumptions or corrections, such as tests of normality and adjustment for multiple comparisons
☐	☒	A full description of the statistical parameters including central tendency (e.g. means) or other basic estimates (e.g. regression coefficient) AND variation (e.g. standard deviation) or associated estimates of uncertainty (e.g. confidence intervals)
☐	☒	For null hypothesis testing, the test statistic (e.g. F, t, r) with confidence intervals, effect sizes, degrees of freedom and P value noted<br>*Give P values as exact values whenever suitable.*
☒	☐	For Bayesian analysis, information on the choice of priors and Markov chain Monte Carlo settings
☒	☐	For hierarchical and complex designs, identification of the appropriate level for tests and full reporting of outcomes
☒	☐	Estimates of effect sizes (e.g. Cohen's d, Pearson's r), indicating how they were calculated

Our web collection on statistics for biologists contains articles on many of the points above.

Software and code

Policy information about availability of computer code

Data collection	No software was used for the collection of data
Data analysis	All source code for image processing is publicly available under an open-source-license at https://github.com/KatherLab and given with the exact commit ID (Supp. file 2: Table S4) for reasons of reproducibility. The tesselation script is available at https://github.com/KatherLab/preprocessing-ng and followed by normalization with the script available at https://github.com/KatherLab/preProcessing. Extraction of CTransPath features was carried out with scripts from https://github.com/KatherLab/marugoto as well as the transformer based Deep Learning model and the heatmaps for explainability used for the study.

For manuscripts utilizing custom algorithms or software that are central to the research but not yet described in published literature, software must be made available to editors and reviewers. We strongly encourage code deposition in a community repository (e.g. GitHub). See the Nature Portfolio guidelines for submitting code & software for further information.

Data

Policy information about availability of data

All manuscripts must include a data availability statement. This statement should provide the following information, where applicable:

- Accession codes, unique identifiers, or web links for publicly available datasets
- A description of any restrictions on data availability
- For clinical datasets or third party data, please ensure that the statement adheres to our policy

The whole slide images, molecular and clinical data for the TCGA cohort are publicly accessible at https://portal.gdc.cancer.gov/ and https://www.cbioportal.org/. For this study we used the MSI status for the TCGA cohort based on the findings of Liu et al. at https://github.com/KatherLab/cancer-metadata/blob/main/tcga/liu.xlsx. The datasets from DACHS and APHP are available from the corresponding author on reasonable request.

Research involving human participants, their data, or biological material

Policy information about studies with human participants or human data. See also policy information about sex, gender (identity/presentation), and sexual orientation and race, ethnicity and racism.

Reporting on sex and gender	Our study has 2,533 samples in total, derived from 3 cohorts. We provide the full information in Table S1
Reporting on race, ethnicity, or other socially relevant groupings	The country of origin for cohort TCGA is the US, for cohort DACHS is Germany, for cohort APHP is France. Information about race was available for TCGA and is shown in Table S1.
Population characteristics	This information is mentioned in the methods.
Recruitment	All studies contain samples which were collected with the intent of performing translational research.
Ethics oversight	We examined anonymized patient samples from several academic institutions in this investigation. TCGA did not require formal ethics approval for a retrospective study of anonymised samples. The use of tissue samples from DACHS was approved by the Ethics committee of the Medical Faculty at Heidelberg University (310/2001). The use of tissue samples from the APHP cohort was approved by local ethics committee of Henri Mondor University Hospital (IRB N° 00011558 ; 2021-123). The overall analysis was approved by the Ethics board of the Medical Faculty of Technical University Dresden under the ID BO-EK-444102022.

Note that full information on the approval of the study protocol must also be provided in the manuscript.

Field-specific reporting

Please select the one below that is the best fit for your research. If you are not sure, read the appropriate sections before making your selection.

☒ Life sciences ☐ Behavioural & social sciences ☐ Ecological, evolutionary & environmental sciences

For a reference copy of the document with all sections, see nature.com/documents/nr-reporting-summary-flat.pdf

Life sciences study design

All studies must disclose on these points even when the disclosure is negative.

Sample size	No formal method exists, we used large samples with similar numbers as previous studies.
Data exclusions	Mentioned in the methods and supplementary Table S1.
Replication	Random seeds set in the code, patient splits for cross validation were saved for reproducibility.
Randomization	Samples were stochastically allocated into different folds and external validation was performed on all external samples. More details can be found in the methods and accompanying code.
Blinding	Not relevant, the performed study is retrospective.

Reporting for specific materials, systems and methods

We require information from authors about some types of materials, experimental systems and methods used in many studies. Here, indicate whether each material, system or method listed is relevant to your study. If you are not sure if a list item applies to your research, read the appropriate section before selecting a response.

Materials & experimental systems

n/a	Involved in the study
☒	☐ Antibodies
☒	☐ Eukaryotic cell lines
☒	☐ Palaeontology and archaeology
☒	☐ Animals and other organisms
☒	☐ Clinical data
☒	☐ Dual use research of concern
☒	☐ Plants

Methods

n/a	Involved in the study
☒	☐ ChIP-seq
☒	☐ Flow cytometry
☒	☐ MRI-based neuroimaging

Plants

Seed stocks	*Report on the source of all seed stocks or other plant material used. If applicable, state the seed stock centre and catalogue number. If plant specimens were collected from the field, describe the collection location, date and sampling procedures.*
Novel plant genotypes	*Describe the methods by which all novel plant genotypes were produced. This includes those generated by transgenic approaches, gene editing, chemical/radiation-based mutagenesis and hybridization. For transgenic lines, describe the transformation method, the number of independent lines analyzed and the generation upon which experiments were performed. For gene-edited lines, describe the editor used, the endogenous sequence targeted for editing, the targeting guide RNA sequence (if applicable) and how the editor was applied.*
Authentication	*Describe any authentication procedures for each seed stock used or novel genotype generated. Describe any experiments used to assess the effect of a mutation and, where applicable, how potential secondary effects (e.g. second site T-DNA insertions, mosiacism, off-target gene editing) were examined.*
